# Pervasive Selection for Cooperative Cross-Feeding in Bacterial Communities

**DOI:** 10.1371/journal.pcbi.1004986

**Published:** 2016-06-17

**Authors:** Sebastian Germerodt, Katrin Bohl, Anja Lück, Samay Pande, Anja Schröter, Christoph Kaleta, Stefan Schuster, Christian Kost

**Affiliations:** 1 Department of Bioinformatics, Friedrich Schiller University Jena, Germany; 2 Research Group Theoretical Systems Biology, Friedrich Schiller University Jena, Germany; 3 Experimental Ecology and Evolution Research Group, Department of Bioorganic Chemistry, Max Planck Institute for Chemical Ecology, Jena, Germany; 4 Research Group Medical Systems Biology, Institute for Experimental Medicine, Christian-Albrechts-University Kiel, Germany; 5 Department of Ecology and Evolution, School of Biology/Chemistry, Osnabrück University, Germany; Rice University, UNITED STATES

## Abstract

Bacterial communities are taxonomically highly diverse, yet the mechanisms that maintain this diversity remain poorly understood. We hypothesized that an obligate and mutual exchange of metabolites, as is very common among bacterial cells, could stabilize different genotypes within microbial communities. To test this, we developed a cellular automaton to model interactions among six empirically characterized genotypes that differ in their ability and propensity to produce amino acids. By systematically varying intrinsic (i.e. benefit-to-cost ratio) and extrinsic parameters (i.e. metabolite diffusion level, environmental amino acid availability), we show that obligate cross-feeding of essential metabolites is selected for under a broad range of conditions. In spatially structured environments, positive assortment among cross-feeders resulted in the formation of cooperative clusters, which limited exploitation by non-producing auxotrophs, yet allowed them to persist at the clusters’ periphery. Strikingly, cross-feeding helped to maintain genotypic diversity within populations, while amino acid supplementation to the environment decoupled obligate interactions and favored auxotrophic cells that saved amino acid production costs over metabolically autonomous prototrophs. Together, our results suggest that spatially structured environments and limited nutrient availabilities should facilitate the evolution of metabolic interactions, which can help to maintain genotypic diversity within natural microbial populations.

## Author Summary

Natural bacterial communities are usually very species-rich and bacterial cells within these communities often exchange metabolites with each other. Whether and to which extent obligate metabolic interactions can contribute to maintaining the observed bacterial diversity, however, is not known. In this study, we address this question computationally, by simulating populations of six different bacterial strains that differ in their requirement to obtain amino acids from the environment as well as their propensity to release other amino acids. By systematically varying key variables such as the cost of metabolite production, the speed with which metabolites diffuse in the environment, as well as the amino acid availability in the environment, we show that a cooperative exchange of essential amino acids is evolutionary stable over a broad range of biologically realistic conditions. In particular spatially structured environments, such as bacterial biofilms, and moderate costs of metabolite production favored metabolic interactions. Finally, our work identifies obligate metabolic interactions as a powerful ecological mechanism to maintain different bacterial genotypes with microbial communities.

## Introduction

Bacteria are ubiquitous and play a fundamental role in sustaining life, for example by driving global bio-geochemical cycles [1, 2]. Natural microbial communities are phylogenetically highly diverse assemblages and, in many cases, consist of several thousand interacting species [3]. Recent advances in next generation sequencing demonstrated that even seemingly identical bacterial species from the same microbial community show an enormous variability on the genomic, epigenetic, metabolic, or phosphoproteome levels [4–7].

However, the enormous diversity that is frequently observed within bacterial communities is difficult to reconcile with natural selection, which predicts competition for local resources should reduce genotypic diversity witin bacterial species. Moreover, also when different bacterial species compete for the same resources only those should be able to survive that are best adapted to utilizing these resources (i.e. competitive exclusion principle) [8, 9]. A variety of mechanistic explanations have been proposed to explain the unexpectedly high diversity within microbial communities. For example, the partitioning of resources [10] or their utilization at differential rates [11] can allow different organisms to coexist in the same environment. Alternatively, the competitive monopoly of particularly dominant species can be prevented by disturbance [12], demographic trade-offs [13], predation [14], or non-transitivity of competitive interactions [15].

Ecological niches are not only generated by the abiotic environment, but also by biotic interactions (e.g. competition and mutualism). Moreover, multiple bacterial strains of the same or different species can coexist when they engage in metabolic interactions such as the cross-feeding of metabolic by-products [16, 17] or the exchange of essential nutrients [18]. In both cases, frequency-dependent selection has been suggested to benefit both partners when rare, thus stabilizing these types of interactions in the long-run [13, 18].

While the release of metabolic by-products is most likely incidental and not selected for, an active investment into the production of costly metabolites such as co-factors or amino acids to benefit other bacterial cells (hereafter *cooperative cross-feeding*) requires explanations concerning the formation and evolutionary stability of such interactions. In particular, it is not clear how these kinds of interactions can be stable against the invasion of types that reap benefits without contributing to the production of the released metabolite. Despite this seeming paradox, cooperative cross-feeding is very common in the microbial world [19–22] and has been shown to readily evolve under laboratory conditions [23, 24].

Several plausible explanations could account for the frequent occurrence of cooperative metabolic interactions among microorganisms. First, the preference of microorganisms to exist in spatially-structured biofilm communities could enhance local feedbacks among producing cells and thus increase reciprocity [25–28]. As a consequence, metabolite-producing genotypes may form clusters, which could help to exclude non-producing genotypes from cooperative benefits [29, 30]. Second, the costs of producing certain metabolites may be off-set by receiving others, for which production costs are saved [31, 32]. This ‘*division-of-labor*’ effect could tip the benefit-to-cost ratio in favor of metabolic cross-feeding. Third, the availability of certain metabolites (e.g. amino acids) in the environment may fluctuate over time. While metabolite-replete conditions may strongly select for the loss of biosynthetic genes and, therefore, favor an uptake from the environment [33], subsequent metabolite depletion could promote cross-feeding among newly-evolved auxotrophic genotypes [34].

**Fig 1:**
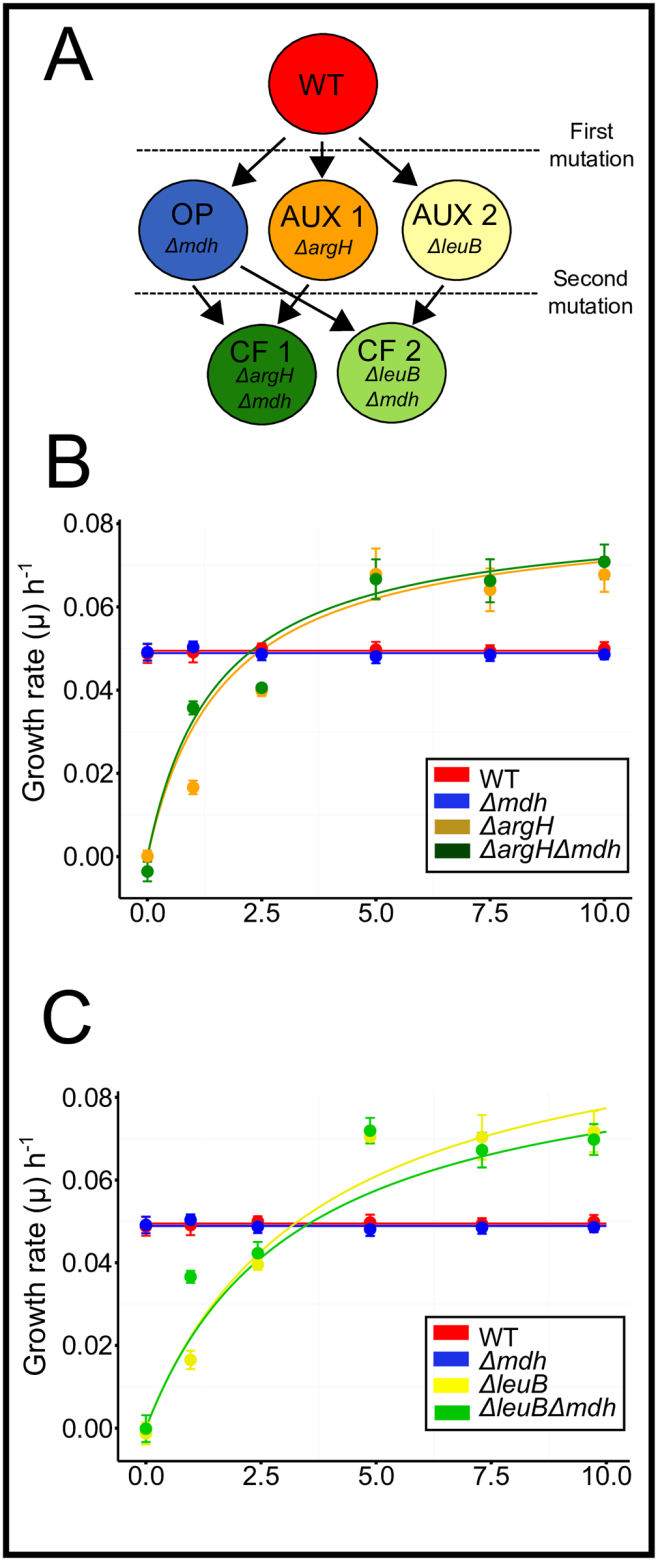
Schematic overview over the focal genotypes and their corresponding growth performance. (A) Genes deleted from the genome of the prototrophic wild type (WT) strain of *Escherichia coli* to yield mutants that are auxotrophic for the amino acids arginine or lysine (AUX1, AUX2), a mutant that overproduces a mixture of both amino acids (OP), and cross-feeding genotypes that are auxotrophic for one, yet produce increased amounts of the respective other amino acid (CF1, CF2). (B,C) Experimentally determined growth performance of all focal genotypes in response to different concentrations of the amino acids (B) arginine and (C) leucine. Amino acids were applied in a mixture of amino acids mimicking the blend of amino acids produced by the overproducer. Growth rate (*μ*) per hour is the experimentally determined Malthusian parameter during 24h of growth. Lines represent fitted Monod kinetics for auxotrophic and cross-feeding genotypes and the calculated mean for the prototrophic (WT) strain (red) as well as the genotype overproducing amino acids (blue). See S1 Text and S3 Fig for further information.

Until now, it remains unclear how much these factors can—singly or in combination—promote the emergence of cooperative cross-feeding of essential metabolites within genetically diverse bacterial populations. Here we address these issues in a cellular automaton modeling approach called CELL-ABC (**Cell**ular **A**utomaton of **B**acterial **C**ross-feeding) to simulate the release of metabolites by bacteria into the surrounding environment as well as their subsequent uptake by other bacterial cells. In this way, the cellular automaton allows us to explicitly analyze spatial effects and emergent population structures. The basis of the simulated bacterial phenotypes is an empirical set of *Escherichia coli* genotypes that differ in their metabolic abilities [18]. These genotypes include: (1) *prototrophic wild type*, (2) a strain producing increased amounts of two amino acids (hereafter ‘*overproducer*’), (3) two genotypes that essentially require one of two amino acids to grow (hereafter ‘*auxotrophs*’), and (4) two genotypes that are auxotrophic for one amino acid, yet produce and release increased amounts of the respective other amino acid into the cell-external environment (hereafter’*cross-feeders*’) (Fig 1).

In this work, we employ CELL-ABC to identify the range of parameters under which cooperative cross-feeding of essential metabolites can persist within bacterial populations and to determine the population-level consequences that arise in terms of genotypic composition and spatial interaction structure. To address these issues, we monitored the dynamics of populations, in which the cost-to-benefit ratio of metabolite cross-feeding, the environmental availability of focal metabolites, and the diffusion of the released metabolites were systematically varied. In particular, the following hypotheses were tested:

1. Metabolic cross-feeding is favored when costs of metabolite production are low.
2. Metabolic cross-feeding interactions are stabilized at low levels of metabolite diffusion.
3. Environments with increased metabolite availabilities favor amino acid auxotrophic genotypes.
4. Obligate metabolic cross-feeding increases the genotypic diversity within bacterial populations.

## Results

### Cooperative cross-feeding is ecologically stable despite increased fitness costs

**Fig 2:**
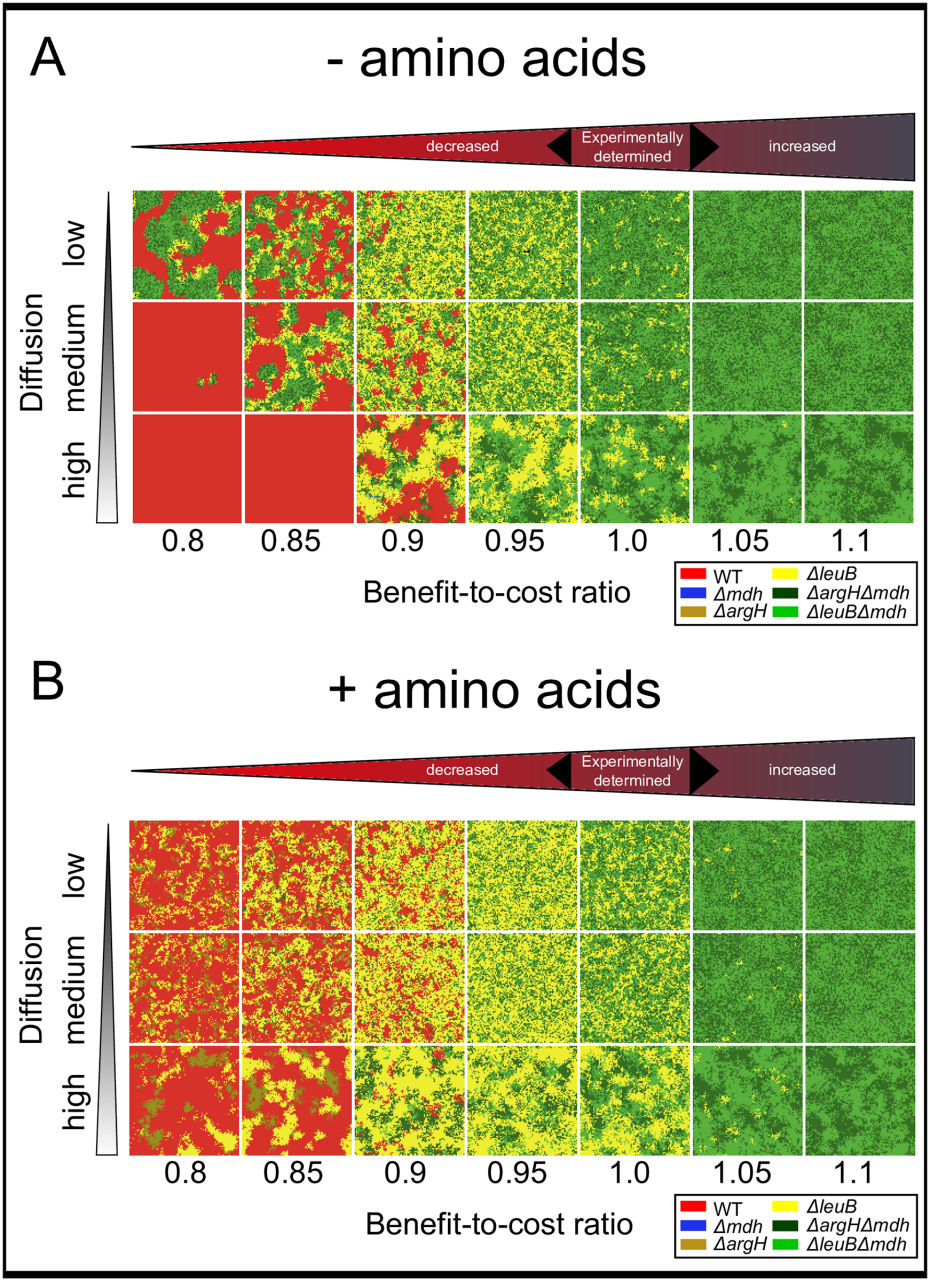
Auxotrophic and cross-feeding genotypes are selectively favored under a broad range of conditions. Shown are representative simulation results after 100 simulation steps. Parameters analyzed include the benefit-to-cost ratio where the experimentally determined values were computationally in- or decreased (x-axes), the degree of metabolite diffusion in the environment (y-axes) ranging from low (structured environment) to high (unstructured environment), and the environmental availability of amino acids (**A,B**) including (**A**) no amino acids are available in the environment, and (**B**) substantial additional availability of amino acids in the environment. Color-code of genotypes: red = wild type, blue = amino acid overproducer, yellow = amino acid auxotrophs (2 types), green = cross-feeding genotypes (2 types).

The fitness of the empirically characterized genotypes was determined in the presence of different amino acid concentrations in the environment (Fig 1B and 1C). While the growth of prototrophic cells (i.e. wild type and the amino acid overproducer) was insensitive to varying amino acid concentrations in the environment, the two cross-feeding types and the two auxotrophs each showed a unique growth response that differed significantly from the one of prototrophic types as well as from each other (ANOVA, *P* < 0.05, *n* = 8, Fig 1B and 1C and S3 Fig). This empirically determined growth response of each of the focal genotypes was defined as a benefit-to-cost ratios (BCRs) of 1. To determine how changes in metabolite production costs would affect the stability of amino acid cross-feeding interactions, the BCR of the simulated genotypes was computationally in- or decreased. Increasing the costs of amino acid overproduction over the experimentally determined values (i.e. *BCR* < 0.8) always led to a stable state, in which wild type cells occupied all available grid-cells. For a BCR between 0.8 and 1.0, mixed populations of wild type, cross-feeding-, and auxotrophic genotypes coexisted, while further decreasing the costs of amino acid production (> 1.0) resulted in a competitive exclusion of prototrophic wild type cells. Finally, when benefits strongly outweighed metabolite production costs (> 1.05), non-cooperating auxotrophs were outcompeted by cross-feeding genotypes (Fig 2A).

**Fig 3:**
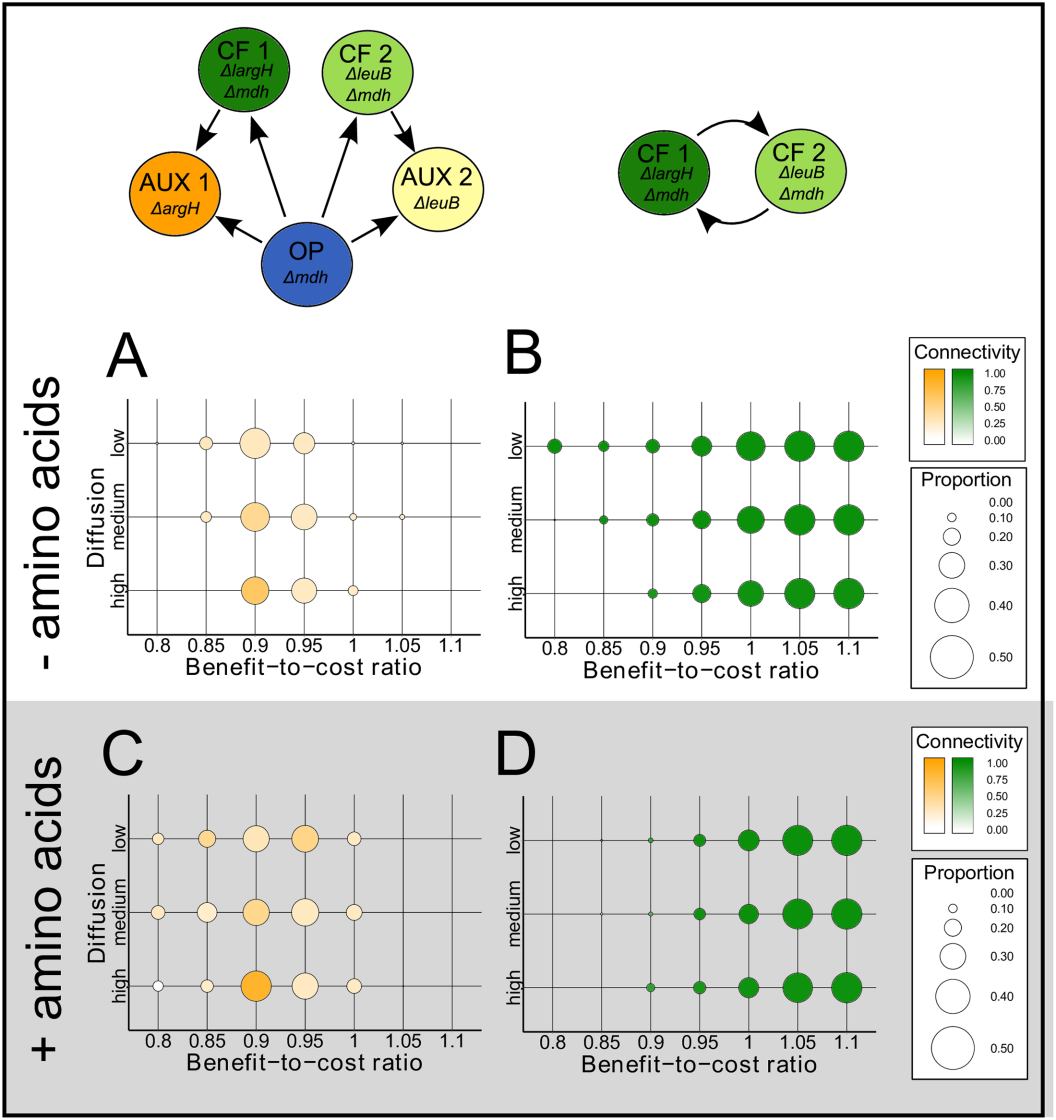
Prevalence of unilateral and bilateral cross-feeding. Sizes of circles represent the mean proportion of (**A, C**) unilateral and (**B, D**) bilateral cross-feeding that included all interactions depicted in the schematic above. Colors of circles represent the mean connectivity between cell types that measures the co-occurrence of genotypes and thus reflects the probability of genotypes to display one or the other type of cross-feeding. 100 simulation runs were analyzed per parameter combination. Simulation results with (**A, B**) no or (**B, D**) a substantial addition of amino acids to the environment are displayed. Parameter combinations that were analyzed in each panel include the benefit-to-cost ratio (x-axes) and the degree of metabolite diffusion in the environment (y-axes) ranging from low (structured environment) to high (unstructured environment).

Characteristic clusters of cross-feeding mutants formed at BCRs ranging between 0.8 and 1.0, which were virtually always flanked by a belt of non-cooperating auxotrophs (Fig 2A). With increasing metabolite production costs, the size of these clusters increased and the thickness of the fringing belt of non-cooperating types decreased. Decreasing the costs of metabolite production generally altered the qualitative distribution of cross-feeding interactions within populations: reciprocal cross-feeding was favored over a unilateral exchange of metabolites (Fig 3A and 3B).

Taken together, these results demonstrate that the costs of amino acid overproduction significantly impacted both the prevalence of cross-feeding genotypes within populations and their spatial distribution. Nevertheless, both unilateral and bilateral cross-feeding was common under a broad range of parameter conditions.

### Reduced metabolite diffusion facilitates the formation of cross-feeding clusters

To determine how the degree of spatial structuring affects metabolic interactions within the resident populations, the number of iterated diffusion steps within a given environment was varied. In this way it was possible to manipulate the spatial distribution of the released metabolites. The results of these analyses showed that a reduced diffusion of amino acids facilitated the formation of clusters consisting of both cross-feeding genotypes (Fig 2A). Although auxotrophic genotypes benefited from the public goods that were released from clusters of cross-feeding genotypes, they occurred exclusively at the periphery of these clusters. This striking pattern was most likely caused by a limited diffusion of amino acids outside of these clusters, which led to a spatial exclusion of non-cooperating auxotrophs from these public goods.

In contrast, when interactions were less localized due to an increased diffusion of metabolites, the benefit auxotrophic mutants gained increased as indicated by the fact that they increasingly accumulated around cross-feeding clusters (BCR = 0.85, Mann–Whitney U test: *P* < 0.05, *n* = 200 and Fig 2A). This characteristic pattern was lost in spatially unstructured environments (mimicking a perfectly mixed environment), in which both auxotrophic- and cross-feeding mutants showed a random spatial distribution (Fig 2A) with no sign of direct metabolic cross-feeding (Fig 3A and 3B).

Taken together, the degree of spatial structuring and thus the access to essential metabolites significantly shaped the composition and spatial distribution of genotypes within the modeled populations. A low diffusion of public goods resulted in the formation of cross-feeding clusters, which were surrounded by non-cooperating auxotrophs that reaped benefits without reciprocating.

### Decoupling obligate interactions affects community structure

To investigate the community-structuring effect of obligate cross-feeding, the requirement for uni- and bilateral cross-feeding was relieved by additionally supplying both essential amino acids to the simulated environments. The added amount of amino acids per grid cell were in the same order of magnitude as the amount of amino acids secreted by overproducing genotypes. Auxotrophic genotypes generally benefited from environmentally available amino acids, as reflected by their increased abundance (Figs 2 and 3). High amino acid concentrations in the environment readily resulted in a total numerical dominance of these genotypes as expected from the growth performance experiments (Fig 1). The observation that also the connectivity of unilateral cross-feeding increased significantly when amino acids were environmentally available (Mantel test: *P*<0.05, *n* = 9999) corroborated the interpretation that auxotrophic genotypes were nutritionally independent under amino acid replete conditions, which resulted in an increased degree of intermixing between different genotypes. Interestingly, an additional supply of amino acids significantly reduced the abundance of cross-feeding genotypes at low BCRs (< 1). Moreover, due to the relief from obligate amino acid exchange under these conditions, the spatial distibution of cross-feeding genotypes was altered (Mantel test: *P* < 0.05, *n* = 9999) with almost no tendency to form clusters (Fig 3).

In sum, externally providing amino acids to the environment decoupled the obligate interactions and thus eliminated the requirements for reciprocal cross-feeding. As a consequence, auxotrophic genotypes that saved amino acid production costs were generally favored over all other genotypes.

### Obligate metabolite cross-feeding increases the genotypic diversity

Finally, the set of simulations conducted was used to systematically investigate the effect of amino acid cross-feeding on the genotypic diversity within the population under a given set of conditions. Starting each simulation run with six different genotypes, which were present in equal numbers and randomly distributed over the grid, the maximal diversity achievable (Shannon-Weaver diversity index H) in the simulated population is 1.792.

**Fig 4:**
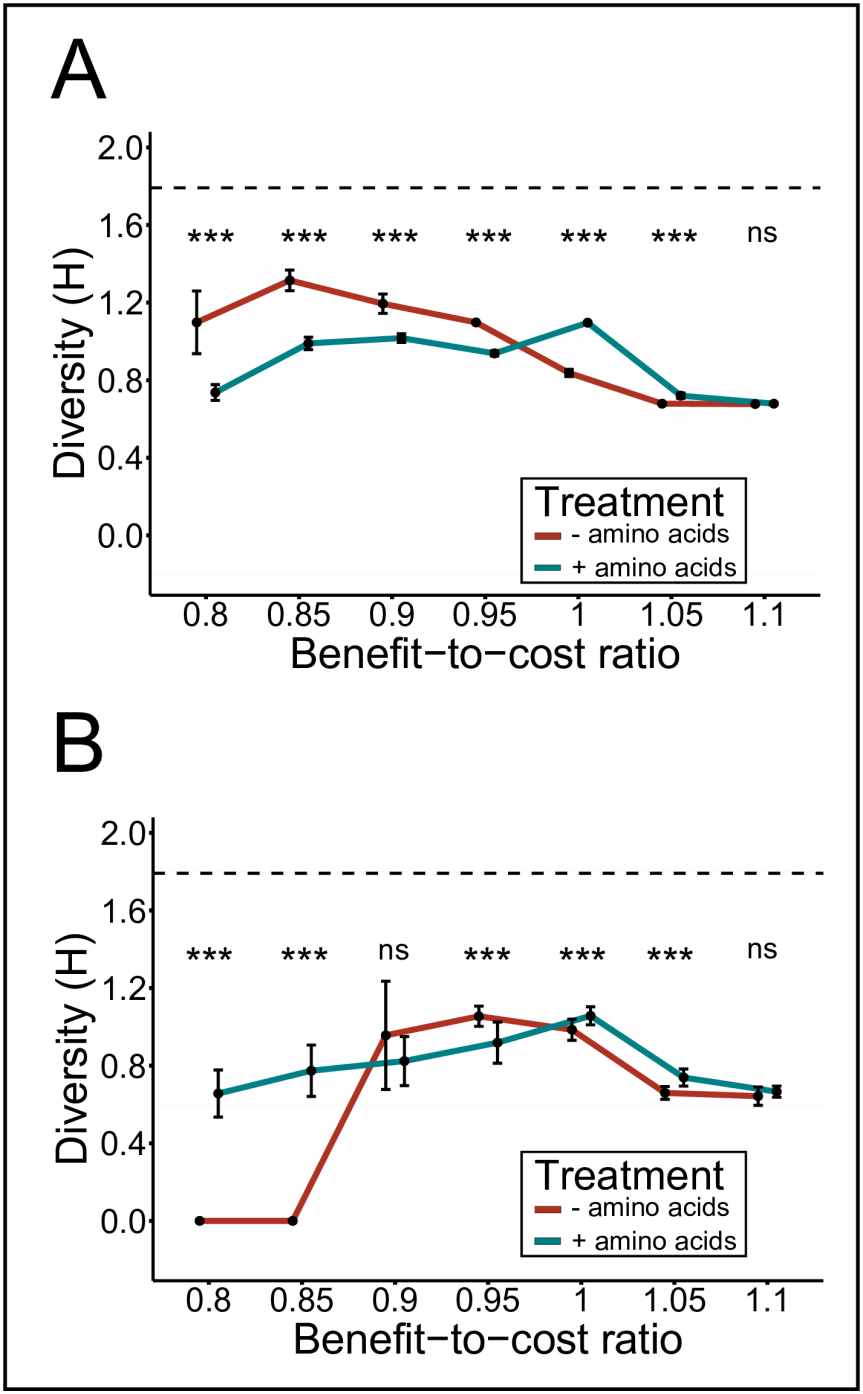
Metabolic cross-feeding increases genotypic diversity within bacterial populations. Mean Shannon-Weaver diversity indices (H ± standard deviation) of simulated populations with varying benefit-to-cost ratios (BCR) are shown. Simulations were performed in the presence (blue line) or absence (red line) of environmentally supplemented amino acids in (**A**) spatially structured (i.e. low diffusion) and (**B**) unstructured environments (i.e. high diffusion). The dashed line indicates the maximally achievable diversity index for six genotypes. Asterisks indicate significant differences between the amino acid supplemented- and unsupplemented environment for a given BCR (FDR-corrected two-sample t-test: *** *P* < 0.001, ns: *P* > 0.05, n = 50).

Investigating the genotypic diversity for scenarios, in which the fitness cost of amino acid overproduction was computationally in- or decreased (i.e. BCR 0.8 − 1.1) relative to experimentally determined genotypes, revealed a bell-shaped diversity distribution in response to increasing BCRs in low diffusion conditions (Fig 4A). The highest diversity (here 1.31, which corresponds to 73% of the maximally achievable diversity) emerged at a BCR of 0.85 (i.e. 15% costs of amino acid overproduction relative to experimentally determined values). Strikingly, an external supply of amino acids to the growth environment significantly lowered diversity levels populations achieved within spatially structured environments (Fig 4A), while in unstructured environments amino acid supplementation had the opposite effect, particularly at very low (i.e. < 0.9 BCR values (Fig 4B)). In the absence of environmentally available amino acids, levels of genotypic diversity showed a strong positive correlation with the prevalence of two-way cross-feeding in the corresponding communities (Spearman’s rank correlation *r* = 0.79, *n* = 14) when levels of metabolite diffusion were reduced. In contrast, amino acid supplementation to unstructured environments benefited auxotrophs and—to a lesser extent—also cross-feeding genotypes that otherwise (in the absence of amino acids) were largely dominated by monocultures of wild type cells (BCR < 0.85). Altogether, these results revealed strong interactive effects between the degree of environmental structure and an increased availability of the required metabolites in the environment on the genotypic diversity of the focal populations.

### Dynamic replacement of metabolic strategies

**Fig 5:**
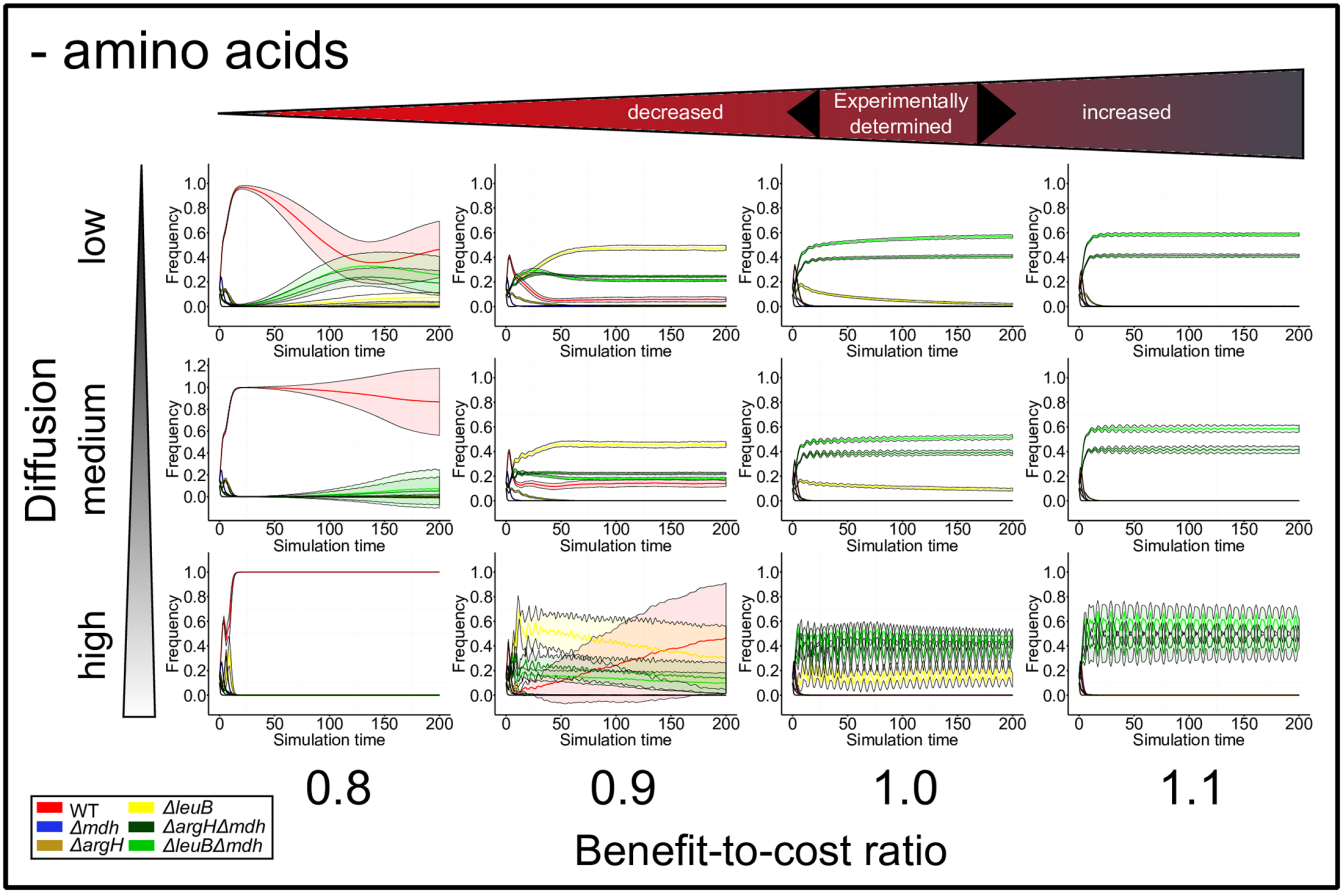
Population dynamics in environments without amino acid supplementation. Repeated simulations (n = 100) are plotted for varying benefit-to-cost ratios (BCR) and degrees of amino acid diffusion (bold line: mean, shaded ribbon: standard deviation). All simulations start with a random distribution of all genotypes and undergo a specific dynamic alternation of genotype frequencies. Depending on the genotype’s strategy, it can repress, facilitate, or even outcompete others (see text for more details). Legend: red = wild type, blue = overproducing genotype, yellow = auxotroph (2 types), green = cross-feeder (2 types).

The simulations were frequently characterized by a non-linear development from the random initial distribution of genotypes to the final steady state. This steady state was qualitatively independent of initial community compositions over a broad range of parameter combinations (S2 Fig). While the abundance of overproducing genotypes always converged immediately to zero, the fraction of the remaining strategies commonly followed a complex pattern (Fig 5). Surprisingly, the abundance of cross-feeders often initially dropped—even for parameter settings that promoted reciprocal cross-feeding in the long run. Here, prototrophic wild type cells and auxotrophic genotypes underwent a short-term increase in their frequency shortly after the simulation started. High abundances of auxotrophic genotypes reduced the overall concentration of amino acids and thus diminished the frequency of cross-feeding genotypes providing the public good. This feedback mechanism damped the increase in the frequency of auxotrophic genotypes. However, random co-localization of complementary cross-feeders in spatially structured environments (i.e. low metabolite diffusion) resulted in fitness values that exceeded wild type levels and rapidly developed into fast-growing clusters of cross-feeders. Their spreading across the grid was often flanked by non-producing and hitch-hiking auxotrophic genotypes. Simultaneously, the fraction of grid cells occupied by wild type declined and usually strategies flew into an oscillating steady state with fluctuating, yet stable patterns (Fig 5). When additional amino acids were available, wild type and auxotrophic mutants were the most dominant genotypes. Their frequencies showed a damped oscillating pattern (S5 Fig). Under these conditions, cross-feeders generally occurred at very low frequencies, except for BCR > 1.

Taken together, cross-feeding interactions drove the dynamic turnover of strategies within metabolically diverse population and promoted spatio-temporal oscillations of genotype frequencies.

## Discussion

Obligate cross-feeding of essential metabolites is very common in the microbial world [19, 21]. The conditions that favor such synergistic interactions and the consequences that result for the structure and composition of the resident microbial community, however, remain poorly understood. Here we address these issues by identifying the range of conditions that maintain metabolic cross-feeding and, thus, genotypic diversity within bacterial populations. Using genetically engineered loss-of-function mutants as empirical basis, we explore how changes of intrinsic (i.e. benefit-to-cost-ratio of metabolic cross-feeding) and extrinsic factors (i.e. environmental amino acids availability, diffusion rate) affect the ecological dynamics within genotypically diverse populations (i.e. six genotypes). Our study revealed that unilateral and bilateral cross-feeding of essential metabolites that are based on the release of these metabolites into the cell-external environment is stably maintained over a broad range of conditions including increased costs of metabolite production and increased diffusion rates. Only when the costs of metabolite production exceeded a certain threshold (i.e. > 20% relative to the experimentally determined values) or environments were perfectly mixed, prototrophic genotypes outcompete all other types present. An environmental availability of amino acids selected for auxotrophic- and against cross-feeding genotypes. Obligate metabolic cross-feeding helped to maintain genotypic diversity in spatially structured environments, while nutrient supplementation to the environment counteracted this effect.

Our analysis of environments that did not contain the limiting resource (i.e. amino acids) identified two conditions under which obligate metabolic cross-feeding maintained genotypic diversity within a bacterial population: First, a high degree of spatial structuring, and second, low production costs of the traded metabolites (Fig 4). These predictions are corroborated by empirical data. For example, *Salmonella enterica* rapidly evolved increased amino acid production rates to support the growth of auxotrophic *E. coli* cells, which in turn produced metabolic waste products *Salmonella* needed to grow [23]. Also in this case, spatial structure was essential for the costly amino acid overproduction mutation to increase in frequency once it had evolved. In contrast, unilateral cross-feeding, in which a receiving genotype is an adaptive mutant that consumes metabolic by-products released by the ancestral donor, does not require spatial structure to allow for coexistence between both partners. A well-documented example of such a cross-feeding polymorphism that emerged and was maintained even in a shaken, liquid environment is the case of acetate-cross-feeders that evolved from glucose-utilizing *Escherichia coli* cells [16, 35]. Since this interaction is likely non-obligatory and does not incur a fitness cost to the producing cell, its maintenance in spatially unstructured environments is also predicted by our model (Fig 2A).

However, what is the ecological mechanism that favored consortia of cross-feeding genotypes in amino acid-deficient, structured environments? Under these conditions, groups of cross-feeding cells that coincidentally co-localized formed cell clusters that enjoyed the benefits of cooperative cross-feeding, which more than compensated for the costs of metabolite overproduction. In the long-run, these cooperative clusters could persist despite the presence of non-producing genotypes, most likely because cross-feeders within these clusters enjoyed the benefits of a cooperative metabolite exchange, which were less available to non-cooperating auxotrophs outside these clusters [36]. These conditions resemble the situation experienced by cells growing in a biofilm, in which principles of spatial self-organization facilitate positive assortment among cross-feeding genotypes [37, 38]. Indeed, both theoretical [39, 40] and experimental studies [30, 41] have previously identified spatial structure as a factor favoring the evolution of cooperative interactions. Oliveira and coworkers [42], however, concluded based on theoretical grounds that problems to find the right complementary genotype in spatially structured environments can also inhibit the evolution of metabolic cross-feeding interactions between genotypes. In contrast to these predictions, our results with six bacterial genotypes that were parametrized using empirical data show that low levels of metabolite diffusion can in fact promote cooperation between complementary cross-feeding genotypes. Experiments using different *E. coli* mutants to test these predictions are currently being performed and will be presented elsewhere.

Simulations with benefit-to-cost ratios between 0.8 and 1.0 (i.e. up to 20% costs to the experimentally determined values) and a low degree of metabolite diffusion revealed specific temporal dynamics. These were characterized by a characteristic alternation of abundances, especially of wild type-, auxotrophic-, and cross-feeding genotypes. First, an increase in the frequency of auxotrophic- and a decline of cross-feeding genotypes was observed to a point, at which the community was not able to sustain more non-cooperating auxotrophic mutants and almost collapsed. Prototrophic wild type genotypes benefited from this situation and thus increased in frequency. This was accompanied by a dramatic decline of genotypic richness in the community and a harmonization of local genotype assemblages. At this point, coincidentally co-localized cross-feeders formed founder populations that subsequently re-populated the grid. Expanding cross-feeding clusters were virtually always flanked by a belt of auxotrophic genotypes. Patches in which auxotrophs persist may thus function as a genetic reservoir, from which cooperative cross-feeding can arise by mutation and spread throughout the population when environmental conditions change.

The local turnover of wild type cells, cross-feeding clusters, and auxotrophic mutants in spatially structured environments combined with the observation that global genotype abundances remained relatively stable, is strikingly reminiscent of a spatial zero-sum game (e.g. the ‘rock-scissor-paper game’). Biological examples include non-transitive competitive networks as displayed by bacteriocin- producing, -resistant, and -sensitive *E. coli* cells (see [43] for a review and [44] for a theoretical study) or the reproductive strategies of small lizards (*Uta stansburiana*) that are associated with color polymorphisms [45].

Our results reveal that environments, in which the essentially required metabolites were not limiting, strongly selected for auxotrophic genotypes (Fig 2). This pattern was independent of the production costs and the diffusion rate of the focal metabolite. This finding is in line with theoretical work showing that cooperation is favored under resource limited conditions [46] and experimental studies demonstrating that auxotrophic mutants of different bacterial species (i.e. *E. coli*, *Acinetobacter baylyi*, and *Bacillus subtilis*) that lack the ability to biosynthesize a certain metabolite gain a selective advantage in environments that contain the corresponding metabolite in sufficient amounts [33, 47]. Also cross-feeding genotypes benefited from increased metabolite availabilities in the environment—albeit this advantage only manifested at higher benefit-to-cost ratios (Fig 3). This was likely due to the fact that even though cross-feeders saved the costs to produce one amino acid, they were still burdened with the investment to produce increased amounts of other amino acids. Only when these production cost were very low, cross-feeders increasingly benefited from environmentally supplemented amino acids as well as the metabolite released by the corresponding other cross-feeder.

Interestingly, our results demonstrate that obligate cross-feeding of essential metabolites can stabilize genotypic richness in microbial communities even above the limits that are predicted by the competitive exclusion principle [8, 9]. According to this theory, the number of different species that can coexist is limited by the number of resources that are available in the same environment. In the case analyzed in this study, all genotypes utilize the same carbon source, yet some of them provide new resources that are essentially required by others to grow. Thus, when amino acids are lacking in the environment, both overproducers and cross-feeders construct the niche that allows other community members (i.e. auxotrophs and cross-feeders) to grow [48, 49]. Conversely, externally providing the required metabolites to genotypically diverse communities uncoupled the obligate metabolic interactions and significantly reduced the genotypic diversity in spatially structured environments. This effect is analogous to the so-called ‘*paradox of enrichment*’ [50]: supplementation of limiting nutrients to an ecosystem does not relax competitive interactions, but intensifies them, by favoring the most competitive species. Originally proposed for interactions between two trophic levels (i.e. predator-prey interactions), nutrient addition has also been shown to destabilize steady states of competitive ecosystems [50]. Thus, our study extends this list by obligate metabolic cross-feeding interactions that are ecologically uncoupled by an environmental nutrient availability. As a consequence, more competitive genotypes will take over, which ultimately leads to a loss of genotypic diversity in the population. Strikingly, experimental nutrient supplementation to soil also resulted in a significantly reduced bacterial diversity [51]. However, future work is necessary to determine whether and to which extend this result was due to the uncoupling of obligate cross-feeding interactions.

A main conclusion that follows from the results of our study is that spatially structured environments that show fluctuating nutrient availabilities should select for a loss of biosynthetic genes when the corresponding metabolites are sufficiently available in the environment, yet favor cooperative cross-feeding when metabolite levels drop below a certain level. Indeed, bacteria usually exist in highly structured environments [52], in which they experience frequent changes in the availability of (essential) nutrients [53, 54] and both uni- and bilateral cross-feeding is common in these bacterial communities [19–22]. Moreover, less than 1% of all bacterial species known are amenable to laboratory cultivation in monoculture [55, 56], yet this fraction can be increased by growing seemingly unculturable bacteria in the presence of other community members [57]. These findings suggest that obligate cross-feeding of essential metabolites could explain the frequently observed difficulties to cultivate natural bacteria isolates under laboratory conditions. The fact that simply the deletion of three different metabolic genes was sufficient to generate the complex patterns of metabolic interdependencies [18] analyzed in this work suggests gene loss is a powerful source of synergistic ecological interactions. Once established, obligate metabolic interactions may intensify in a ‘*black-queen*’-type race [58], in which locally interacting partners loose additional metabolic functions that are compensated by other community members. Over time, this process should lead to increasingly intertwined metabolic interactions within microbial communities, whose dynamics will most likely be also determined by the key parameters identified in this study: i) degree of spatial structuring, ii) benefit-to-cost ratio, and iii) environmental availability of exchanged nutrients.

Given the enormous fitness advantages that result from the different metabolic interactions a question arises: What maintains prototrophic genotypes in the long-run? By losing the ability to produce certain metabolites, auxotrophic genotypes as well as the type of cross-feeders analyzed here and in [18] trade their metabolic autonomy against an immediate fitness advantage. As a consequence, the reproduction of these types becomes contingent on an environmental supply of the required nutrient, reflecting the dilemma of specialization versus flexibility. Assuming the environmental conditions to which bacterial metapopulations are exposed change frequently, prototrophic bacteria should be globally maintained, because some local patches feature conditions under which they are selectively favored. In this case, prototrophic genotypes would serve as generalist dispersal unit that can found new populations, in which newly-emerged adaptive loss of function mutants can thrive. This scenario is consistent with prototrophic bacterial pathogens such as *Pseudomonas fluorescens* that opportunistically infect the lung of cystic fibrosis patients and—due to increased metabolite concentrations in the sputum—rapidly evolve amino acid auxotrophies [59, 60].

Our results indicate that obligate metabolic interactions represent a strong ecological force to stabilize a range of different genotypes, which can help to maintain genotypic diversity within microbial populations and communities. Especially spatial structure with limited metabolite diffusion favored cooperative cross-feeding via local feedbacks that excluded less efficient cooperators or, non-cooperating auxotrophic genotypes. Our model predicts that biofilms (i.e. highly structured environments with very limited metabolite diffusion) and environments that frequently fluctuate in their nutrient availability should generally select for cooperative cross-feeding. Taken together, by implementing biologically realistic parameter values, our model suggests that mutualistic cross-feeding interactions between different genotypes should readily evolve in microbial communities.

## Methods

The description of CELL-ABC basically follows the ODD-protocol, which was developed for describing individual- and agent-based models [61, 62]. Non-applicable sections of the protocol were omitted.

### CELL-ABC model description (ODD protocol)

#### Purpose

This model aims at simulating metabolic exchange among bacteria to identify the conditions under which cross-feeding is evolutionarily stable despite competition with metabolically autonomous genotypes (i.e. amino acid overproducing- and prototrophic genotypes) as well as with non-cooperating auxotrophs.

#### Entities, state variables, and scales

In our model, cells of *Escherichia coli* strains are represented by the lattice sites (grid-cells) of a Cartesian grid. The grid has periodic boundary conditions and was determined to dimensions of 100*x*100 cells as previously determined in a pilot study (see S4A Fig). Grid-cells can either be free or occupied by a strategy. There are six types of strategies, which differ in their fundamental metabolic properties: prototrophic wild type, a genotype overproducing the two amino acids (arginine (Arg) and leucine (Leu)), two genotypes that are auxotrophic for Arg and Leu (non-cooperators), and two cross-feeding genotypes (i.e. cooperators) that are auxotrophic for one, yet produce increased amounts of the other amino acid. These six genotypes represent a functional subset of the model system used by Pande *et al.* (2014) [18] (Fig 1). Additionally, each grid-cell has a list of concentrations of the two focal amino acids Arg and Leu representing the amount of amino acids currently available in this grid-cell. Thus, a grid-cell represents both an individual (i.e. characterized by its strategy and metabolic properties) as well as the environment (i.e. the local concentration of amino acids that is temporally available).

Each genotype that occupies a grid-cell has a fitness value that results from the local availability of amino acids (see Input section). The fitness calculation procedure (see Submodel section) of overproducing genotypes is extended by a benefit-to-cost ratio (BCR) parameter to account for the costs of amino acid overproduction or the benefits stemming from the saving of amino acid overproduction costs.

One update step in the model represents 10 minutes in real-time. Secretion and growth processes derived from experimental results are normalized to this time interval. All simulations were evaluated after 100 simulation steps (see S4B Fig). The diffusion parameter simulates slow, fast, or a homogeneous distribution of interchanged metabolites (thus simulating the effect of spatial structure). In this way, bacterial growth can be simulated either under normal diffusion conditions or in a homogeneous (i.e. spatially unstructured) environment. S1 Table provides an overview over the state variables and parameters used.

#### Process overview and scheduling

At every discrete time step, each process (S1 Fig) is carried out simultaneously by all grid-cells. One update step proceeds as follows: grid-cells with mutants carrying overproduction mutations release amino acids by updating their list of amino acid concentrations (see submodel *Metabolite Secretion*). Then diffusion is applied to the amino acids in each grid-cell (see submodel *Diffusion*). All occupied grid-cells then calculate their fitness value (see submodel *Fitness Calculation*). Grid-cells with fitness values equal to zero (e.g. to a lack of essential amino acids) were assumed to be not viable. These grid-cells were set to empty and thus are excluded from the fitness-based contest. The last process of each update step is the spreading of genotypes (i.e. replication), in which all grid-cells participate in a fitness-based contest in their Moore neighborhood and with a weighted probability adopt the strategy of the neighbor with the highest fitness-value (see submodel *Spreading*).

#### Initialization

Initially, all six focal genotypes are randomly assigned to grid-cells with a probability of 15%, so that all types are equi-abundant. The remaining 10% of cells are left empty. Different initial starting frequencies of cross-feeding genotypes revealed no qualitative differences in the final community composition over a broad range of parameter combinations (S2 Fig). Fitness values and all local amino acid concentrations are set to 0.

#### Input

The model has two main interfaces, in which experimental data have been implemented: i) growth experiments analyzing the dependence of the genotypes’ growth rates (Fig 1B and 1C) in response to different amino acid concentrations in the environment and ii) biosensor experiments revealing the amount of amino acids produced by each genotypes per unit time (see S1 Text). Diffusion coefficients of amino acids were obtained from the literature [63, 64].

#### Output

A parameter to quantitatively describe the co-occurrence of genotypes (i.e. potential cross-feeding interactions) was calculated from the mean minimal Euclidean distance (MMED) for each combination of mutant strategies. The calculations were performed for each grid-cell to 30% of randomly chosen non-empty cells. Since a random initial distribution of six different mutants results in an expected MMED of 1/6, each deviation from this value is caused by interactions among genotypes (i.e. fitness-based contests). The MMED was calculated after 100 time-steps from 50 independent simulation runs for each parameter combination. Beyond this number of iterations, all grid-cells were usually occupied by the same genotypes (e.g. all wild type) or the ratio of remaining strategies did not change significantly. Finally the MMED values were transformed to a measure of connectivity:

$$\begin{array}{c} \text{connectivity}=1-\frac{\bar{\text{MMED}}}{{\text{MMED}}_{max}} \end{array}$$ (1)

Thus, cells with a low MMED indicate a high connectivity and thus represent a high degree of co-occurrence among genotypes. Connections, in which one or both genotypes had gone extinct during simulations, were set to a connectivity value of zero. For further analysis, metabolic cross-feeding interaction were classified as *unilateral* and *bilateral* depending on the directionality of metabolic exchange. Unilateral cross-feeding occurs between overproducing-, auxotrophic-, and cross-feeding mutants, while bilateral interactions are restricted to reciprocal cross-feeding interactions between two cross-feeding genotypes. Spatial co-occurrence of genotypes was used to estimate the abundance and directionality of cross-feeding.

### 0.1 Submodels

#### Metabolite secretion

All genotypes carrying the overproduction mutation produced increased amounts of amino acids. While the overproducer secretes increased amounts of Arg and Leu, the cross-feeding genotypes solely produce either Arg or Leu. The amount of secreted amino acid per cell and time interval was calculated from biosensor experiments (see S1 Text). The local concentrations of amino acids are stored in concentration lists that are assigned to each grid-cell.

#### Diffusion

In CELL-ABC, diffusion of amino acids is modeled as proposed by Grajdeanu [65]. The local amino acid concentrations of both aminoacids (i.e. Arg and Leu) were synchronously updated for all cells. We chose this diffusion model over NetLogo’s built-in diffusion routine, because we anticipated that the stability of cross-feeding consortia should depend on their position relative to each other and on small differences in environmental amino acid concentrations. In contrast to NetLogo’s built-in diffusion routine, the model by Grajdaenu discriminates between orthogonal and diagonal neighbors of a grid-cell and thus allows to more realistically represent diffusion processes within a discrete grid.

The chosen *diffusion-radius* regulates how often the diffusion calculation is repeated at each update step, thereby determining the rate of diffusion of amino acids in relation to a grid-cell’s update step. Increasing diffusion radii flatten the gradient and extend the spatial distribution of amino acids. This increases the amount of amino acids the focal cell receives from more, and potentially more diverse, neighboring patches, while the local competition of neighboring cells is maintained. The cumulative local concentration of amino acids is lower when diffusion radii are large as compared to smaller diffusion radii that show steeper gradients.

#### Fitness calculation

For each grid-cell, a fitness value is calculated depending on the local availability of amino acids. For this, results of the growth performance experiments were used (i.e. amino acid concentration-dependent growth curves as determined for wild type, amino acid auxotrophs, overproducers, and cross-feeding genotypes) (see Input section and S1 Text for further details). Thus the fitness of a single genotype is determined by its own strategy, the qualitative and quantitative composition of its neighborhood (i.e. amino acid donors or auxotrophs), and diffusion parameters. Fitness values of overproducing and cross-feeding genotypes can additionally be in- or decreased relative to experimentally determined values to computationally in- or decrease the costs of metabolite overproduction. The resulting parameter ‘benefit-to-cost-ratio’ (BCR) is set to 1 for the experimentally characterized model genotypes. BCR values < 1 represent cases, in which the costs for amino acid overproduction were computationally increased over the empirical example, while values > 1 simulate cases in which amino acids were less costly to overproduce.

The fitness of non-overproducing genotypes (i.e. wild type and auxotrophs) is calculated by:

$$\begin{array}{c} f_{wt}=f_{aux}=\frac{V_{max}*c_{localAA}}{K_{m}+c_{localAA}} \end{array}$$ (2)

Parameter *V*_*max*_ and *K*_*m*_ are derived from *in vitro* experiments for each genotype (see Supplement S1 Text and S3 Fig). The local amount of amino acid is given by *c*_*local*_. For all genotypes that produce increased amount of amino acids (i.e. overproducer and cross-feeders) the BCR parameter is added to alter the benefit to cost ratio:

$$\begin{array}{c} f_{op}=f_{cf}=BCR*\frac{V_{max}*c_{localAA}}{K_{m}+c_{localAA}} \end{array}$$ (3)

#### Spreading

For each cell the neighbor with the highest fitness value in its Moore neighborhood (including itself) is identified that has not yet spread in this time step. A weighted, fitness-based contest is performed to simulate competition, making it possible for the cell with the lower fitness value to keep its settings. In the contest, a random number between 0 and 1 is drawn from a uniform distribution. If it is below $\frac{f_{p}}{f_{p}+f_{n}}$, where *f*_*p*_ is the fitness value of the focal cell and *f*_*n*_ is the fitness value of the fittest neighbor, the grid-cell keeps its settings, otherwise the fittest neighbor overgrows the less fit cell. If there is more than one fittest neighbor, one type is chosen randomly from a uniform distribution.

## Supporting Information

S1 Table Table of state variables and parameters. (PDF)

S1 Fig UML of CELL-ABC representing the order of the main simulation processes. Diagram of the basic processes and procedures of CELL-ABC model.
(TIF)

S2 Fig Varying initial fraction of cross-feeding genotypes. Repeated simulations (n = 200) are plotted for varying initial fractions (5% to 95%) of cross-feeding genotypes (i.e. CF1 and CF2) in the community. Simulations were run for low (A and C) and high (B and D) diffusion conditions, both in the absence (A and B) and presence (C and D) of an environmental supply of amino acid. Under none of the four treatments analyzed did the initial community-level proportion of cross-feeding genotypes qualitatively affect their final frequency in the community.
(TIF)

S3 Fig Experimentally determined growth parameters of all six genotypes. A Monod kinetic was fitted to the growth of all genotypes. Based on this, the growth parameters (**A**) *V*_*max*_ and (**B**) *K*_*M*_ were determined for wild type (WT), the overproducer (Δ*mdh*, OP), the arginine auxotroph (ΔargH, AUX 1), the leucine auxotroph (ΔleuB, AUX 2), as well as the two cross-feeders (Δ*argH*Δ*mdh*, CF 1, Δ*leuB*Δ*mdh*, CF 2). Different letters indicate significant differences between groups (**A**: Kruskal-Wallis test followed by a Tamhanes post-hoc test: *P* < 0.05, *n* = 8, **B**: two-way ANOVA followed by a SNK post-hoc test: *P* < 0.05, *n* = 8).
(TIF)

S4 Fig Simulated genotype fractions at different grid dimensions and durations of simulation runs. Fractions of simulated genotypes after (**A**) 100 simulation steps using grids of different dimensions (*n* = 15) or (**B**) on a grid with the dimensions 100*x*100 grid cells after simulations of a different duration (*n* = 15). Both parameters were varied to identify the optimal grid size and simulation duration that would yield representative genotype distributions while minimizing computational costs. This preliminary analysis revealed a grid size of ≥ 30*x*30 cells and ≥ 100 simulation steps was required for the planned analysis.
(TIF)

S5 Fig Population dynamics in environments with amino acid supplementation. Repeated simulations (n = 100) are plotted for varying benefit-to-cost ratios (BCR) and degrees of amino acid diffusion (bold line: mean, shaded ribbon: standard deviation). All simulations start with a random distribution of all genotypes and undergo a specific dynamic alternation of genotypes frequencies. Depending on the genotype’s strategy, it can repress, facilitate, or even outcompete others (see text for more details). Legend: red = wild type, blue = overproducing genotype, yellow = auxotroph (2 types), green = cross-feeder (2 types).
(TIF)

S1 Text Laboratory experiments. (PDF)

## References

[1] Conrad R. Soil microorganisms as controllers of atmospheric trace gases (H2, CO, CH4, OCS, N2O, and NO). Microbiological Reviews. 1996;60(4):609–640. Available from: http://mmbr.asm.org/content/60/4/609.short. 8987358 10.1128/mr.60.4.609-640.1996 PMC239458

[2] Falkowski PG, Fenchel T, Delong EF. The microbial engines that drive Earth’s biogeochemical cycles. Science (New York, NY). 2008 5;320(5879): 1034–1039. Available from: http://www.ncbi.nlm.nih.gov/pubmed/18497287. 10.1126/science.1153213 18497287

[3] Torsvik V, Sørheim R, Goksøyr J. Total bacterial diversity in soil and sediment communities—a review. Journal of Industrial Microbiology. 1996;17:170–178. Available from: http://link.springer.com/article/10.1007/BF01574690. 10.1007/BF01574690

[4] Parfrey LW, Knight R. Spatial and temporal variability of the human microbiota. Clinical Microbiology and Infection. 2012;18(SUPPL. 4):8–11. 22647040 10.1111/j.1469-0691.2012.03861.x

[5] Cho I, Blaser MJ. The human microbiome: at the interface of health and disease. Nature Reviews Genetics. 2012;13(4):260–70. 10.1038/nrg3182 22411464 PMC3418802

[6] Su C, Lei L, Duan Y, Zhang KQ, Yang J. Culture-independent methods for studying environmental microorganisms: methods, application, and perspective. Applied Microbiology and Biotechnology. 2012;93(3):993–1003. Available from: http://link.springer.com/10.1007/s00253-011-3800-7. 10.1007/s00253-011-3800-7 22189863

[7] Shokralla S, Spall JL, Gibson JF, Hajibabaei M. Next-generation sequencing technologies for environmental DNA research. Molecular Ecology. 2012;21(8):1794–1805. Available from: http://doi.wiley.com/10.1111/j.1365-294X.2012.05538.x. 10.1111/j.1365-294X.2012.05538.x 22486820

[8] Gause G. Experimental analysis of Vito Volterra’s mathematical theory of the struggle for existence. Science. 1934;79(2036): 16–17. Available from: http://faculty.jsd.claremont.edu/dmcfarlane/bio146mcfarlane/papers/Gause_paramecium.pdf. 10.1126/science.79.2036.16-a 17821472

[9] Hardin G. The competitive exclusion principle. Science. 1960;131(3409): 1292–1297. Available from: http://www.esf.edu/efb/schulz/seminars/hardin.pdf. 14399717 10.1126/science.131.3409.1292

[10] Wilson M, Lindow S. Coexistence among epiphytic bacterial populations mediated through nutritional resource partitioning. Applied and Environmental Microbiology. 1994;60(12):4468–4477. Available from: http://aem.asm.org/content/60/12/4468.short. 16349462 10.1128/aem.60.12.4468-4477.1994 PMC202007

[11] Inouye R, Tilman D. Convergence and divergence of old-field plant communities along experimental nitrogen gradients. Ecology. 1988;69(4):995–1004. Available from: http://www.jstor.org/stable/1941254. 10.2307/1941254

[12] Buckling A, Kassen R, Bell G, Rainey P. Disturbance and diversity in experimental microcosms. Nature. 2000;408(6815): 961–964. Available from: http://www.nature.com/nature/journal/v408/n6815/abs/408961a0.html. 10.1038/35050080 11140680

[13] Turner PE, Souza V, Lenski RE. Tests of ecological mechanisms promoting the stable coexistence of two bacterial genotypes. Ecology. 1996;77(7):2119–2129. 10.2307/2265706

[14] Chao L, Levin B, Stewart F. A complex community in a simple habitat: an experimental study with bacteria and phage. Ecology. 1977;58(2):369–378. Available from: http://www.jstor.org/stable/1935611. 10.2307/1935611

[15] Kerr B, Riley M, Feldman M, Bohannan B. Local dispersal promotes biodiversity in a real-life game of rock–paper–scissors. Nature. 418(6894): 171–174. Available from: http://www.nature.com/nature/journal/v418/n6894/abs/nature00823.html. 12110887 10.1038/nature00823

[16] Helling RBR, Vargas CNC, Adams J. Evolution of *Escherichia coli* during growth in a constant environment. Genetics. 1987;116(3): 349–358. Available from: http://www.genetics.org/content/116/3/349.short. 3301527 10.1093/genetics/116.3.349 PMC1203146

[17] Rosenzweig RF, Sharp RR, Treves DS, Adams J. Microbial evolution in a simple unstructured environment: Genetic differentiation in *Escherichia coli*. Genetics. 1994;137(4):903–917. Available from: http://www.genetics.org/content/137/4/903.short. 7982572 10.1093/genetics/137.4.903 PMC1206068

[18] Pande S, Merker H, Bohl K, Reichelt M, Schuster S, de Figueiredo LF, et al Fitness and stability of obligate cross-feeding interactions that emerge upon gene loss in bacteria. The ISME Journal. 2014 5;8(5):953–962. Available from: http://www.ncbi.nlm.nih.gov/pubmed/24285359. 10.1038/ismej.2013.211 24285359 PMC3996690

[19] Schink B. Synergistic interactions in the microbial world. Antonie Van Leeuwenhoek. 2002;81(1–4): 257–261. Available from: http://link.springer.com/article/10.1023/A:1020579004534. 10.1023/A:1020579004534 12448724

[20] Morris BEL, Henneberger R, Huber H, Moissl-Eichinger C. Microbial syntrophy: Interaction for the common good. FEMS Microbiology Reviews. 2013;37(3):384–406. 10.1111/1574-6976.12019 23480449

[21] Sieuwerts S, de Bok FaM, Hugenholtz J, van Hylckama Vlieg JET. Unraveling microbial interactions in food fermentations: from classical to genomics approaches. Applied and Environmental Microbiology. 2008 8;74(16):4997–5007. Available from: http://www.pubmedcentral.nih.gov/articlerender.fcgi?artid=2519258&tool=pmcentrez&rendertype=abstract. 10.1128/AEM.00113-08 18567682 PMC2519258

[22] Seth EC, Taga ME. Nutrient cross-feeding in the microbial world. Frontiers in Microbiology. 2014;5(350). 10.3389/fmicb.2014.00350 PMC4086397 25071756

[23] Harcombe W. Novel cooperation experimentally evolved between species. Evolution. 2010; 64(7): 2166–2172. Available from: http://www.ncbi.nlm.nih.gov/pubmed/20100214. 20100214 10.1111/j.1558-5646.2010.00959.x

[24] Poltak SR, Cooper VS. Ecological succession in long-term experimentally evolved biofilms produces synergistic communities. The ISME Journal. 2011;5(3):369–378. Available from: http://www.nature.com/doifinder/10.1038/ismej.2010.136. 10.1038/ismej.2010.136 20811470 PMC3105725

[25] Doebeli M, Knowlton N. The evolution of interspecific mutualisms. Proceedings of the National Academy of Sciences of the United States of America. 1998;95(15):8676–8680. 10.1073/pnas.95.15.8676 9671737 PMC21135

[26] Foster KR, Wenseleers T. A general model for the evolution of mutualisms. Journal of Evolutionary Biology. 2006;19(4):1283–1293. 10.1111/j.1420-9101.2005.01073.x 16780529

[27] Bull JJ, Rice WR. Distinguishing mechanisms for the evolution of co-operation. Journal of Theoretical Biology. 1991;149(1):63–74. 10.1016/S0022-5193(05)80072-4 1881147

[28] Kreft JU. Biofilms promote altruism. Microbiology. 2004;150(8):2751–2760. 10.1099/mic.0.26829-0 15289571

[29] Verbruggen E, El Mouden C, Jansa J, Akkermans G, Bücking H, West Sa, et al Spatial structure and interspecific cooperation: theory and an empirical test using the mycorrhizal mutualism. The American Naturalist. 2012 5;179(5): E133–E146. Available from: http://www.ncbi.nlm.nih.gov/pubmed/22504548. 10.1086/665032 22504548

[30] Momeni B, Waite AAJ, Shou W. Spatial self-organization favors heterotypic cooperation over cheating. eLife. 2013 1;2:e00960 Available from: http://www.pubmedcentral.nih.gov/articlerender.fcgi?artid=3823188&tool=pmcentrez&rendertype=abstract http://elifesciences.org/content/2/e00960.abstract. 10.7554/eLife.00960 24220506 PMC3823188

[31] Wahl L, Gerrish P, Saika-Voivod I. Evaluating the impact of population bottlenecks in experimental evolution. Genetics. 2002;162(2): 961–971. Available from: http://www.genetics.org/content/162/2/961.short. 12399403 10.1093/genetics/162.2.961 PMC1462272

[32] Harvey E, Heys J, Gedeon T. Quantifying the effects of the division of labor in metabolic pathways. Journal of Theoretical Biology. 2014;360:222–242. Available from: http://www.ncbi.nlm.nih.gov/pubmed/25038317. 10.1016/j.jtbi.2014.07.011 25038317 PMC4162874

[33] D’Souza G, Waschina S, Pande S, Bohl K, Kaleta C, Kost C. Less is more: selective advantages can explain the prevalent loss of biosynthetic genes in bacteria. Evolution. 2014; 68(9): 2559–2570. Available from: http://www.ncbi.nlm.nih.gov/pubmed/24910088. 10.1111/evo.12468 24910088

[34] Wintermute E, Silver P. Dynamics in the mixed microbial concourse. Genes & Development. 2010; 24(23): 2603–2614. Available from: http://genesdev.cshlp.org/content/24/23/2603.short. 10.1101/gad.1985210 21123647 PMC2994034

[35] Treves DS, Manning S, Adams J. Repeated evolution of an acetate-crossfeeding polymorphism in long-term populations of *Escherichia coli*. Molecular Biology and Evolution. 1998; 15(7):789–797. 10.1093/oxfordjournals.molbev.a025984 9656481

[36] Pande S, Kaftan F, Lang S, Svatos A, Germerodt S, Kost C. Privatization of cooperative benefits stabilizes mutualistic cross-feeding interactions in spatially structured environments. The ISME Journal. 2015; 10(6): 1413–1423. 26623546 10.1038/ismej.2015.212 PMC5029186

[37] van Gestel J, Weissing FJ, Kuipers OP, Kovács AT. Density of founder cells affects spatial pattern formation and cooperation in *Bacillus subtilis* biofilms. The ISME Journal. 2014; 8(10): 2069–2079. Available from: http://www.ncbi.nlm.nih.gov/pubmed/24694715. 24694715 10.1038/ismej.2014.52 PMC4184017

[38] Xavier JaB, Martinez-Garcia E, Foster KR. Social evolution of spatial patterns in bacterial biofilms: when conflict drives disorder. The American Naturalist. 2009;174(1):1–12. 10.1086/599297 19456262

[39] Nowak Ma, Bonhoeffer S, May RM. Spatial games and the maintenance of cooperation. Proceedings of the National Academy of Sciences. 1994 5;91(11):4877–4881. Available from: http://www.pnas.org/cgi/doi/10.1073/pnas.91.11.4877. 10.1073/pnas.91.11.4877 PMC43892 8197150

[40] Estrela S, Brown SP. Metabolic and demographic feedbacks shape the emergent spatial structure and function of microbial communities. PLoS Computational Biology. 2013 1;9(12):e1003398 Available from: http://www.pubmedcentral.nih.gov/articlerender.fcgi?artid=3873226&tool=pmcentrez&rendertype=abstract. 10.1371/journal.pcbi.1003398 24385891 PMC3873226

[41] Drescher K, Nadell CD, Stone Ha, Wingreen NS, Bassler BL. Solutions to the public goods dilemma in bacterial biofilms. Current Biology. 2014;24(1):50–55. 10.1016/j.cub.2013.10.030 24332540 PMC3935403

[42] Oliveira NM, Niehus R, Foster KR. Evolutionary limits to cooperation in microbial communities. Proceedings of the National Academy of Sciences. 2014;111(50): 17941–17946. Available from: http://www.pnas.org/content/111/50/17941. 10.1073/pnas.1412673111 PMC4273359 25453102

[43] Riley Ma, Wertz JE. Bacteriocin diversity: Ecological and evolutionary perspectives. Biochimie. 2002;84(5–6):357–364. 10.1016/S0300-9084(02)01421-9 12423779

[44] Neumann G, Schuster S. Continuous model for the rock-scissors-paper game between bacteriocin-producing bacteria. Journal of Mathematical Biology. 2007;54(6):815–846. 10.1007/s00285-006-0065-3 17457587

[45] Sinervo B, Lively C. The rock-paper-scissors game and the evolution of alternative male strategies. Nature. 1996;380(21):240–243. Available from: http://www.tb.ethz.ch/education/model/RPS/sinervo.pdf. 10.1038/380240a0

[46] Ferreira FF, Campos PRa. Multilevel selection in a resource-based model. Physical Review E—Statistical, Nonlinear, and Soft Matter Physics. 2013;88(1):1–4. 10.1103/PhysRevE.88.014101 23944591

[47] Zamenhof S, Eichhorn H. Study of microbial evolution through loss of biosynthetic functions: establishment of “defective”mutants. Nature. 1967;216(5114): 456–458. Available from: http://europepmc.org/abstract/MED/4964402. 10.1038/216456a0 4964402

[48] Odling-Smee F, Laland K, Feldman W. Niche Construction. The American Naturalist. 1996;147(4):641–648. 10.1086/285870 31868536

[49] Day RL, Laland KN, Odling-Smee J. Rethinking Adaptation. Perspectives in Biology and Medicine. 2003;46(1):80–95. Available from: http://muse.jhu.edu/content/crossref/journals/perspectives_in_biology_and_medicine/v046/46.1day.html. 10.1353/pbm.2003.0003 12582272

[50] Rosenzweig ML. Paradox of enrichment: destabilization of exploitation ecosystems in ecological time. Science (New York, NY). 1971;171(969):385–387. 10.1126/science.171.3969.385 5538935

[51] Campbell BJ, Polson SW, Hanson TE, Mack MC, Schuur EaG. The effect of nutrient deposition on bacterial communities in Arctic tundra soil. Environmental Microbiology. 2010;12(7):1842–1854. 10.1111/j.1462-2920.2010.02189.x 20236166

[52] Tolker-Nielsen T, Molin S. Spatial Organization of Microbial Biofilm Communities. Microbial Ecology. 2000;40(2):75–84. Available from: http://www.springerlink.com/content/595x9jmkt19q32m9/∖nhttp://www.ncbi.nlm.nih.gov/entrez/query.fcgi?cmd=Retrieve&db=PubMed&dopt=Citation&list_uids=11029076. 11029076 10.1007/s002480000057

[53] Savageau M. *Escherichia coli* habitats, cell types, and molecular mechanisms of gene control. The American Naturalist. 1983;122(6):732–744. Available from: http://www.jstor.org/stable/2460914. 10.1086/284168

[54] van Elsas JD, Semenov AV, Costa R, Trevors JT. Survival of *Escherichia coli* in the environment: fundamental and public health aspects. The ISME Journal. 2011;5(2):173–183. Available from: http://www.nature.com/doifinder/10.1038/ismej.2010.80. 10.1038/ismej.2010.80 20574458 PMC3105702

[55] Amann R, Ludwig W, Schleifer K. Phylogenetic identification and in situ detection of individual microbial cells without cultivation. Microbiological Reviews. 1995;59(1):143–169. Available from: http://mmbr.asm.org/content/59/1/143.short. 7535888 10.1128/mr.59.1.143-169.1995 PMC239358

[56] Hugenholtz P. Exploring prokaryotic diversity in the genomic era. Genome Biology. 2002;3(2):1–8. Available from: http://www.biomedcentral.com/content/pdf/gb-2002-3-2-reviews0003.pdf. 10.1186/gb-2002-3-2-reviews0003 PMC139013 11864374

[57] Kaeberlein T, Lewis K, Epstein S. Isolating “uncultivable” microorganisms in pure culture in a simulated natural environment. Science. 2002;296(5570): 1127–1129. Available from: http://www.sciencemag.org/content/296/5570/1127.short. 10.1126/science.1070633 12004133

[58] Morris JJ, Lenski RE, Zinser ER. The black queen hypothesis: Evolution of dependencies through adaptive gene loss. mBio. 2012;3(2):1–7. Available from: http://mbio.asm.org/content/3/2/e00036-12.short. 10.1128/mBio.00036-12 PMC3315703 22448042

[59] Barth aL, Pitt TL. Auxotrophic variants of *Pseudomonas aeruginosa* are selected from prototrophic wild-type strains in respiratory infections in patients with cystic fibrosis. Journal of Clinical Microbiology. 1995;33(1):37–40. 7699062 10.1128/jcm.33.1.37-40.1995 PMC227875

[60] Thomas SR, Ray A, Hodson ME, Pitt TL. Increased sputum amino acid concentrations and auxotrophy of *Pseudomonas aeruginosa* in severe cystic fibrosis lung disease. Thorax. 2000;55(9):795–797. 10.1136/thorax.55.9.795 10950901 PMC1745865

[61] Grimm V, Berger U, Bastiansen F, Eliassen S, Ginot V, Giske J, et al A standard protocol for describing individual-based and agent-based models. Ecological Modelling. 2006;198(1–2):115–126. Available from: http://linkinghub.elsevier.com/retrieve/pii/S0304380006002043http://www.sciencedirect.com/science/article/pii/S0304380006002043. 10.1016/j.ecolmodel.2006.04.023

[62] Grimm V, Berger U, DeAngelis DLD, Polhill JG, Giske J, Railsback SF. The ODD protocol: A review and first update. Ecological Modelling. 2010 9;221(23):2760–2768. Available from: http://linkinghub.elsevier.com/retrieve/pii/S030438001000414Xhttp://www.sciencedirect.com/science/article/pii/S030438001000414X. 10.1016/j.ecolmodel.2010.08.019

[63] Longsworth L. Diffusion measurements, at 25, of aqueous solutions of amino acids, peptides and sugars. Journal of the American Chemical Society. 1953;1483(1937):5705–5709. Available from: ttp://pubs.acs.org/doi/abs/10.1021/ja01118a065. 10.1021/ja01118a065

[64] Wu Y, Ma P, Liu Y, Li S. Diffusion coefficients of L-proline, L-threonine and L-arginine in aqueous solutions at 25°C. Fluid Phase Equilibria. 2001;186(1–2): 27–38. Available from: http://www.sciencedirect.com/science/article/pii/S0378381201003557. 10.1016/S0378-3812(01)00355-7

[65] Grajdeanu, A. Modeling Diffusion in a Discrete Environment. In: George Mason University Technical Report Series; 2007. p. 1–5. Available from: http://cs.gmu.edu/~agrajdea/papers/grajdeanu07modeling.pdfhttp://scholar.google.com/scholar?hl=en&btnG=Search&q=intitle:MODELING+DIFFUSION+IN+A+DISCRETE+ENVIRONMENT#0.

