# Supplementary material for: Pervasive Selection for Cooperative Cross-Feeding in Bacterial Communities
Source: PLoS Comput Biol. 2016 Jun 17;12(6):e1004986. doi: 10.1371/journal.pcbi.1004986 (PMC4912067; doi:10.1371/journal.pcbi.1004986)
Supplement: S1 Table — (PDF) [file pcbi.1004986.s001.pdf]

**Table 1. Table of state variables and parameters.**

| State variables | Brief description |
|---|---|
| $C[\cdot]$ | List of current amino acid concentrations. |
| *aux* | Auxotrophy mutations ($AUX1, AUX2, CF1, CF2$). |
| *op* | Overproduction mutation ($OP, CF1, CF2$). |
| *free?* | Boolean variable indicating whether the grid-cell is free or inhabited. |
| $f$ | Fitness |
| **Parameters** | **Description** |
| $BCR$ | Measure of *benefit-to-cost ratio* of amino acid uptake. |
| *diffusion-radius* | Amount of diffusion steps per update step. |
| *adding-feed?* | Boolean variable enabling additional supply of essential amino acids Arg and Leu. |
| $D[\cdot]$ | List of diffusion coefficients of the two amino acids [1,2]. |
| $d$ | Grid-cell diameter |

# References


[1] Longsworth L. Diffusion measurements, at 25, of aqueous solutions of amino acids, peptides and sugars. Journal of the American Chemical Society. 1953;1483(1937):5705–5709. Available from: `http://pubs.acs.org/doi/abs/10.1021/ja01118a065`.

[2] Wu Y, Ma P, Liu Y, Li S. Diffusion coefficients of L-proline, L-threonine and L-arginine in aqueous solutions at 25 C. Fluid Phase Equilibria. 2001;186:27–38. Available from: `http://www.sciencedirect.com/science/article/pii/S0378381201003557`.
