# Supplementary figures and images for: Pervasive Selection for Cooperative Cross-Feeding in Bacterial Communities
Source: PLoS Comput Biol. 2016 Jun 17;12(6):e1004986. doi: 10.1371/journal.pcbi.1004986 (PMC4912067; doi:10.1371/journal.pcbi.1004986)

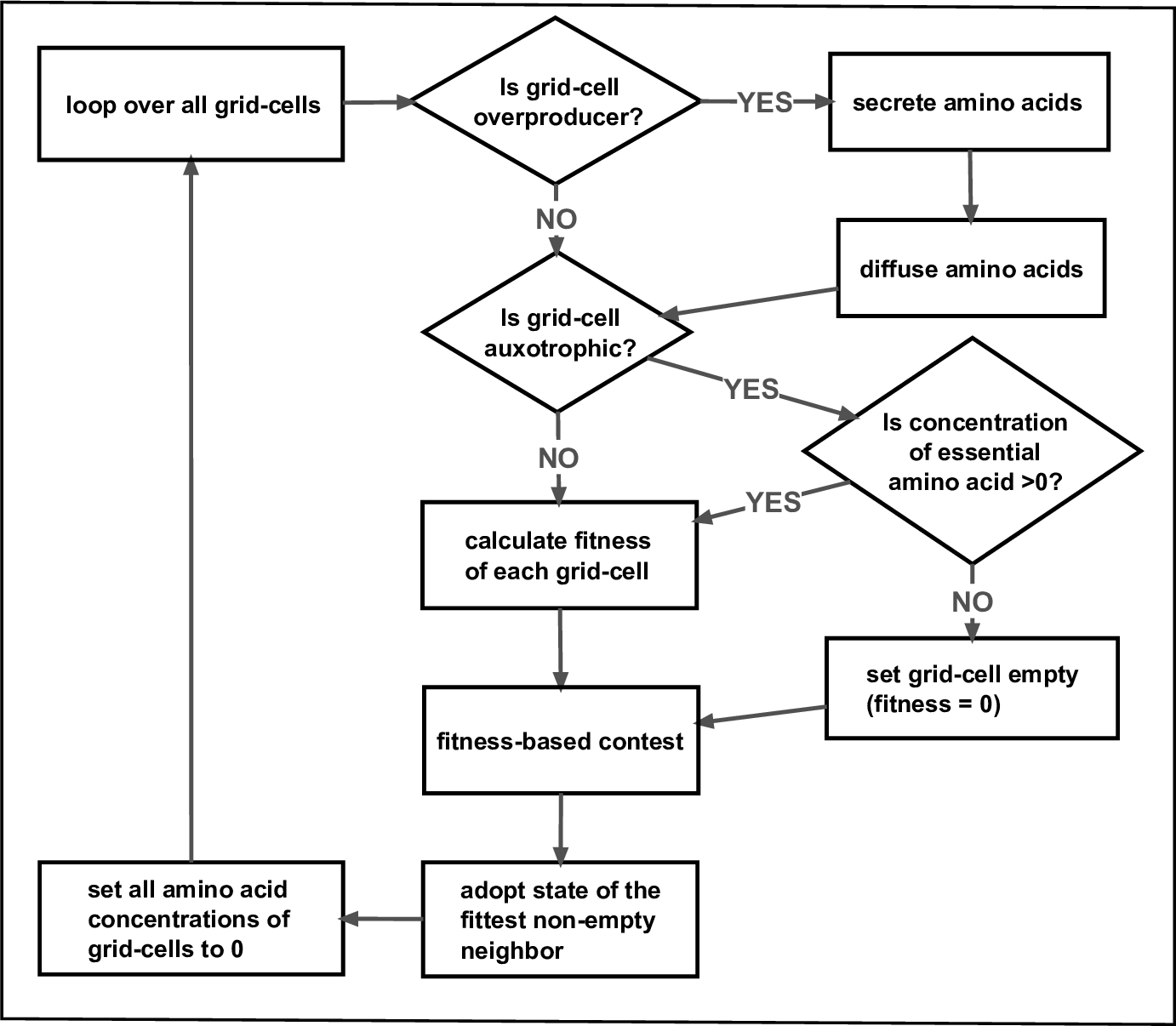

Supplement: S1 Fig — Diagram of the basic processes and procedures of CELL-ABC model. (TIF) [file pcbi.1004986.s002.tif]

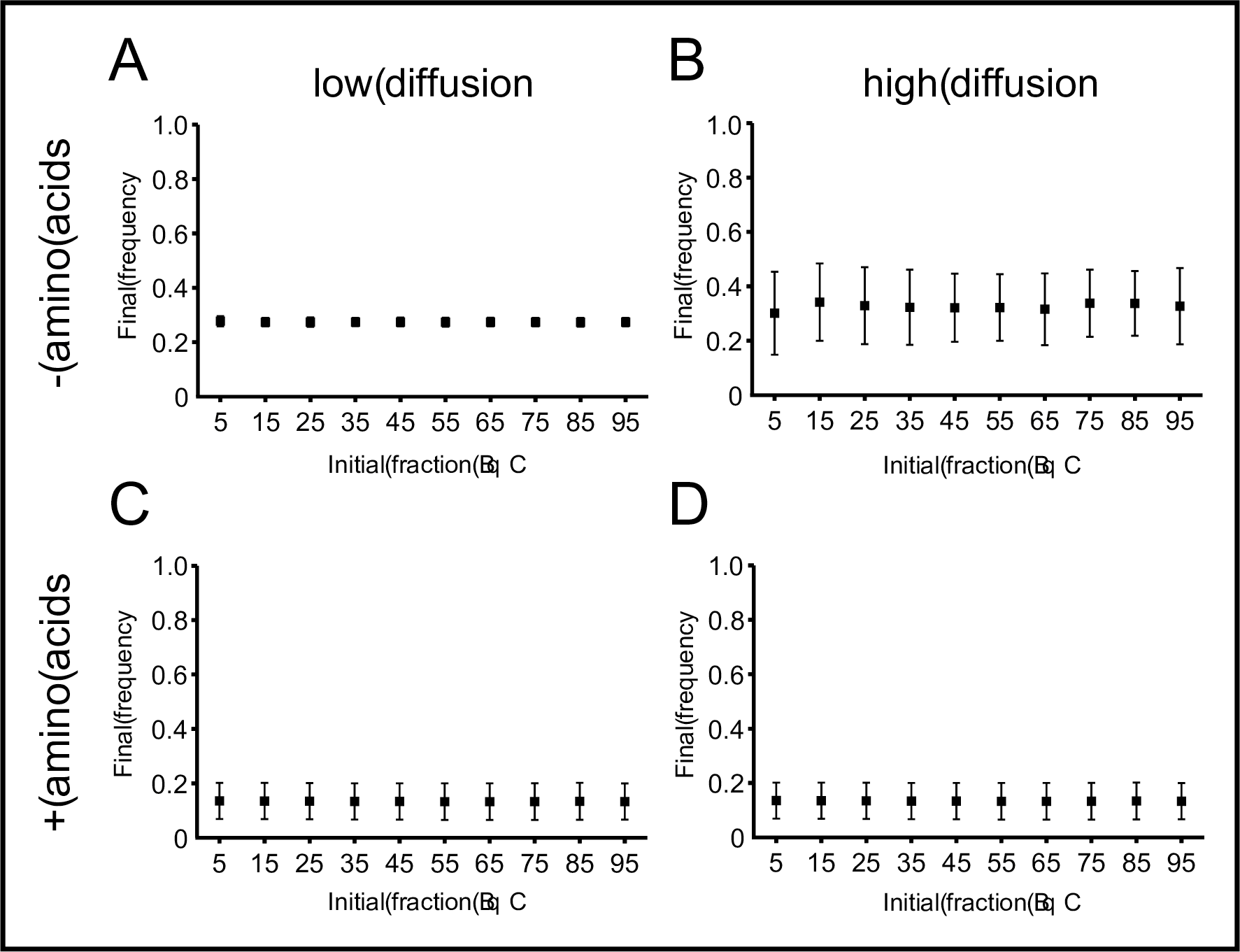

Supplement: S2 Fig — Repeated simulations (n = 200) are plotted for varying initial fractions (5% to 95%) of cross-feeding genotypes (i.e. CF1 and CF2) in the community. Simulations were run for low (A and C) and high (B and D) diffusion conditions, both in the absence (A and B) and presence (C and D) of an environmental supply of amino acid. Under none of the four treatments analyzed did the initial community-level proportion of cross-feeding genotypes qualitatively affect their final frequency in the community. (TIF) [file pcbi.1004986.s003.tif]

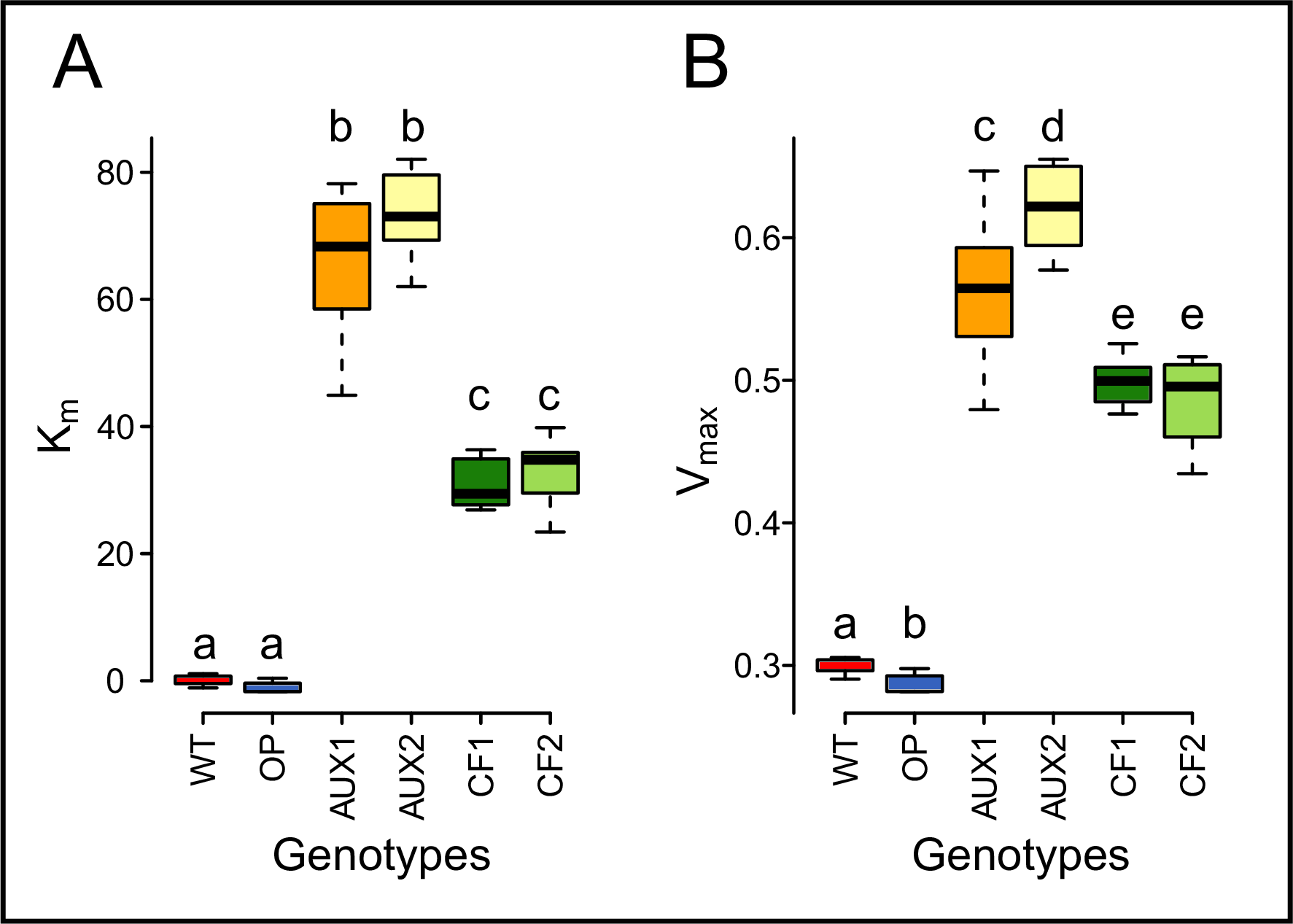

Supplement: S3 Fig — A Monod kinetic was fitted to the growth of all genotypes. Based on this, the growth parameters (A) Vmax and (B) KM were determined for wild type (WT), the overproducer (Δmdh, OP), the arginine auxotroph (ΔargH, AUX 1), the leucine auxotroph (ΔleuB, AUX 2), as well as the two cross-feeders (ΔargHΔmdh, CF 1, ΔleuBΔmdh, CF 2). Different letters indicate significant differences between groups (A: Kruskal-Wallis test followed by a Tamhanes post-hoc test: P < 0.05, n = 8, B: two-way ANOVA followed by a SNK post-hoc test: P < 0.05, n = 8). (TIF) [file pcbi.1004986.s004.tif]

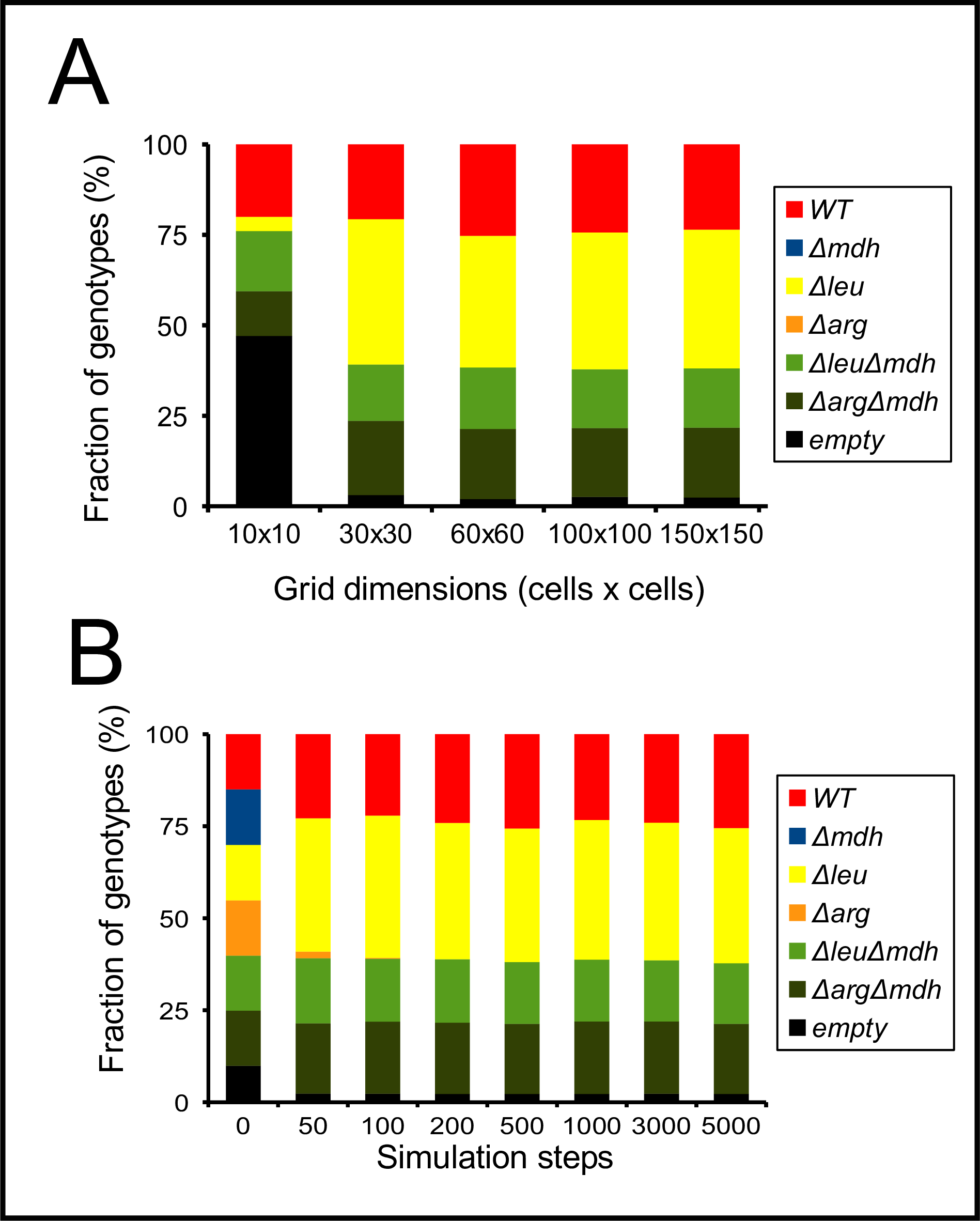

Supplement: S4 Fig — Fractions of simulated genotypes after (A) 100 simulation steps using grids of different dimensions (n = 15) or (B) on a grid with the dimensions 100x100 grid cells after simulations of a different duration (n = 15). Both parameters were varied to identify the optimal grid size and simulation duration that would yield representative genotype distributions while minimizing computational costs. This preliminary analysis revealed a grid size of ≥ 30x30 cells and ≥ 100 simulation steps was required for the planned analysis. (TIF) [file pcbi.1004986.s005.tif]

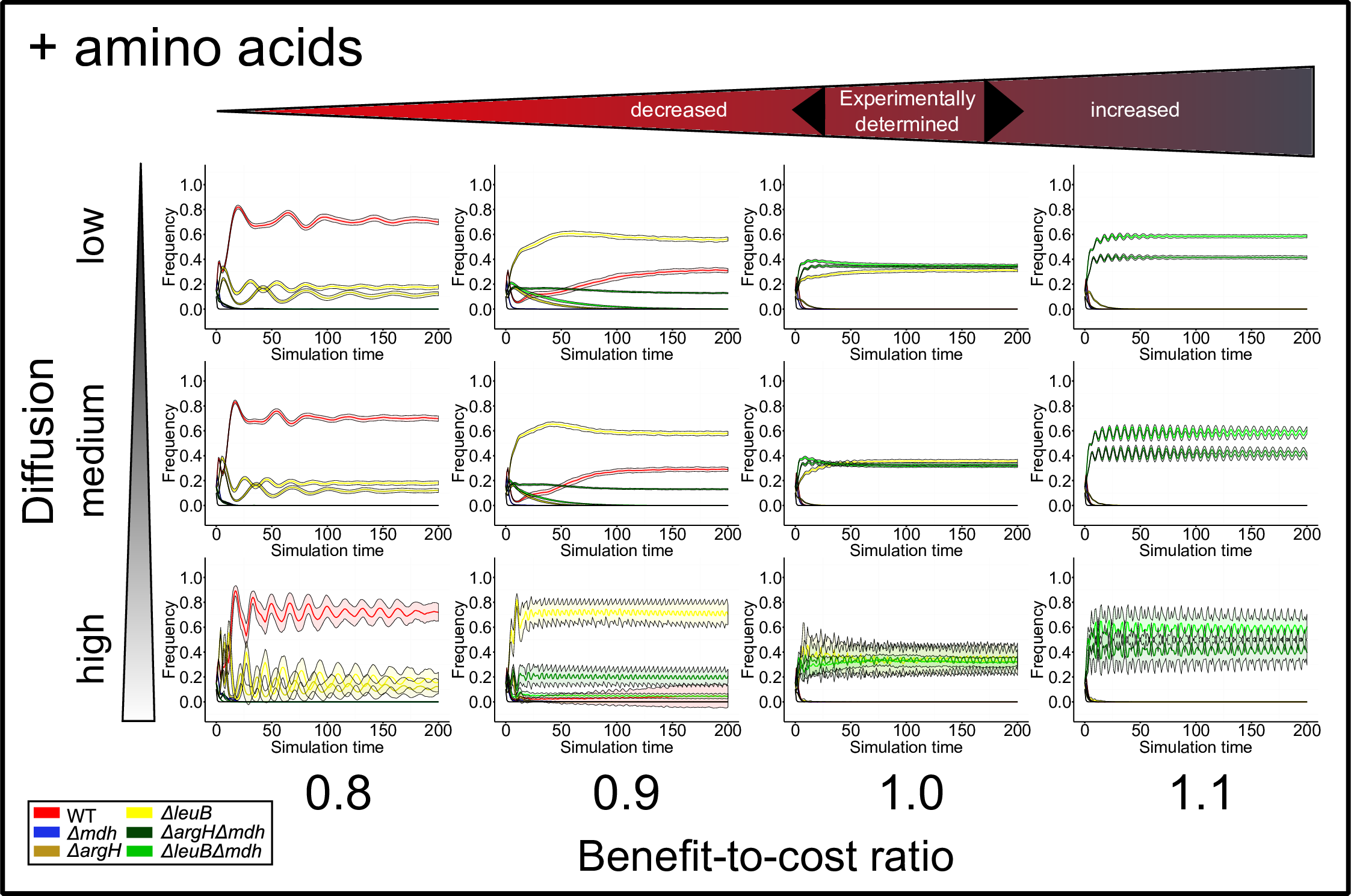

Supplement: S5 Fig — Repeated simulations (n = 100) are plotted for varying benefit-to-cost ratios (BCR) and degrees of amino acid diffusion (bold line: mean, shaded ribbon: standard deviation). All simulations start with a random distribution of all genotypes and undergo a specific dynamic alternation of genotypes frequencies. Depending on the genotype’s strategy, it can repress, facilitate, or even outcompete others (see text for more details). Legend: red = wild type, blue = overproducing genotype, yellow = auxotroph (2 types), green = cross-feeder (2 types). (TIF) [file pcbi.1004986.s006.tif]
