# Supplementary material for: Pervasive Selection for Cooperative Cross-Feeding in Bacterial Communities
Source: PLoS Comput Biol. 2016 Jun 17;12(6):e1004986. doi: 10.1371/journal.pcbi.1004986 (PMC4912067; doi:10.1371/journal.pcbi.1004986)
Supplement: S1 Text — (PDF) [file pcbi.1004986.s007.pdf]

Genotypes used and culture conditions. *Escherichia coli* genotypes used were constructed as described [1] and *E. coli* $BW25113$ served as prototrophic wild type. Cultures were generally incubated at 30 $^{\circ}C$ and all experiments were performed in minimal medium for *Azospirillum brasilense* (MMAB) [2] without biotin and using fructose ($5gl^{-1}$) instead of malate as carbon source. If necessary, arginine (Arg) and leucine (Leu) were added (100 μM) to the medium of precultures.

Amino-acid dependent growth experiments. To experimentally determine the growth rate the six focal strains reached at different amino acid concentrations, overnight cultures of all genotypes were diluted to an optical density of 0.1 at 600 nm (OD_{600nm}). Subsequently, 10μl of the diluted culture was inoculated into 1 ml MMAB medium containing different concentrations of an amino acid mixture (0μM, 20μM, 50μM, 100μM, 150μM, and 200μM) that corresponded to the relative composition of the amino acid mixture produced by Δmdh [1]. To estimate population sizes, cultures were plated on LB agar after 0 h and 24 h of incubation and the number of colony forming units (CFU) were determined for both time points. Afterwards, growth rate (μ) was calculated: $\mu = ln\frac{CFU_{(24h)}ml^{-1}}{CFU_{(0h)}ml^{-1}}$. Growth rate measurements were replicated eight times for every genotype. Both auxotrophic and cross-feeding mutants showed a significant growth response to increasing amino acid availabilities in the medium (one-way ANOVA: $P < 0.005$, n=8), while neither wild type nor the overproducing mutant responded in this way (one-way ANOVA: $P > 0.05$, n=8). Standard curves of growth rate versus focal amino acid concentration in the medium were obtained by fitting Monod kinetics to both parameters. The two kinetic parameters K_M and V_{max} were calculated using the $IC50$ toolkit ($www.ic50.tk$). Both parameters differed significantly between prototrophic types (i.e. overproducer and wild type), auxotrophs and cross-feeders. The mean growth rate of both wild type and overproducer were calculated and the resulting value used in the following modeling processes.

Amino acid quantification using biosensors. In order to estimate amino acid production levels of all six focal genotypes, donor strains were individually co-inoculated together with an *E. coli* mutant that was auxotrophic for either Arg ($\Delta argH$) or Leu ($\Delta leuB$) (initial ratio $1:1$, 5μl 0.1 OD_{600nm} each). After 0 h and 24 h of incubation, cultures were plated on LB agar plates that did or did not contain kanamycin ($50\mu gml^{-1}$) to estimate the population size of both donor and recipient strains. The net count of colony-forming units (CFU ml^{-1}) of amino acid biosensors was determined by subtracting the CFU count at 0 h from the value determined after 24 h. The resulting value was used to estimate the amount of amino acids the respective donor genotype had produced [3]. The molar concentration of amino acids produced was estimated by plotting the growth rate data of recipient strains on the standard curve generated in the experiment above. This experiment was replicated four times.

References

[1] Pande S, Merker H, Bohl K, Reichelt M, Schuster S, de Figueiredo LF, et al. Fitness and stability of obligate cross-feeding interactions that emerge upon gene loss in bacteria. The ISME Journal. 2014 May;8(5):953–62.

[2] Vanstockem M, Michiels K, Vanderleyden J, Van Gool aP. Transposon mutagenesis of *Azospirillum brasilense* and *Azospirillum lipoferum*: Physical analysis of Tn5 and Tn5-Mob insertion mutants. Applied and Environmental Microbiology. 1987;53(2):410–415.

[3] Bertels F, Merker H, Kost C. Design and characterization of auxotrophy-based amino acid biosensors. PLoS ONE. 2012 Jan;7(7):e41349.
